# Digital Immune Gene Expression Profiling Discriminates Allergic Rhinitis Responders from Non-Responders to Probiotic Supplementation

**DOI:** 10.3390/genes10110889

**Published:** 2019-11-04

**Authors:** Nicholas P. West, Annabelle M. Watts, Peter K. Smith, Ping Zhang, Isolde Besseling-van der Vaart, Allan W. Cripps, Amanda J. Cox

**Affiliations:** 1 School of Medical Science, Griffith University, Southport, QLD 4215, Australia; 2 Menzies Health Institute Queensland, Griffith University, Southport, QLD 4215, Australia; 3 School of Medicine, Griffith University, Southport, QLD 4215, Australia; 4 Queensland Allergy Services, Southport, QLD 4215, Australia; 5 Winclove Probiotics B.V., 1032 LB Amsterdam, The Netherlands

**Keywords:** nanostring, allergic rhinitis, probiotics, responders, non-responders, immunoinformatics, mucosal immunology

## Abstract

Probiotic supplementation for eight weeks with a multi-strain probiotic by individuals with allergic rhinitis (AR) reduced overall symptom severity, the frequency of medication use and improved quality of life. The purported mechanism of action is modulation of the immune system. This analysis examined changes in systemic and mucosal immune gene expression in a subgroup of individuals, classified as either responders or non-responders based on improvement of AR symptoms in response to the probiotic supplement. Based on established criteria of a beneficial change in the mini-rhinoconjunctivitis quality of life questionnaire (mRQLQ), individuals with AR were classified as either responders or non-responders. Systemic and mucosal immune gene expression was assessed using nCounter PanCancer Immune Profiling (Nanostring Technologies, Seattle, WA, USA) kit on blood samples and a nasal lysate. There were 414 immune genes in the blood and 312 immune genes in the mucosal samples expressed above the limit of detection. Unsupervised hierarchical clustering of immune genes separated responders from non-responders in blood and mucosal samples at baseline and after supplementation, with key T-cell immune genes differentially expressed between the groups. Striking differences in biological processes and pathways were evident in nasal mucosa but not blood in responders compared to non-responders. These findings support the use of network approaches to understand probiotic-induced changes to the immune system.

## 1. Introduction

Probiotic supplements continue to receive attention as a strategy which may promote positive health outcomes in a range of disease states. For allergic rhinitis (AR), evidence that probiotics can produce clinically meaningful improvements in rhinitis symptoms is mixed [1,2] and may be confounded by issues related to study design. Using a two-stage adaptive trial design [3] we have shown that daily probiotic supplementation improved mini-rhinoconjunctivitis quality of life questionnaire (mRQLQ) scores and reduced the overall severity of symptoms and the frequency of medication use in seasonal AR [4]. The assessment of immune differences between trial participants classified as “responders” and “non-responders” to probiotics may reveal key mucosal immune mechanisms underpinning the clinical effects of these supplements. New digital gene expression technology such as the Nanostring nCounter System is overcoming the limitation of accessing sufficient mucosal material to characterize the immunopathology of AR [5], and is an amplification-independent technique based on hybridization and direct digital counting that allows the simultaneous assessment of multiple targets for consideration of responses in the context of a biological network [6]. To examine the effect of probiotics on systemic and mucosal immunity, we utilized NanoString digital immune gene expression profiling in blood and nasal lavage/ nasal brushing samples in a subset of responders and non-responders from an eight-week supplementation study [4].

## 2. Methods

A Simon two-stage design was utilised for the primary clinical trial [3,4]. In this study a total of 44 patients with clinically confirmed positive allergic response to Bermuda grass (couch) consumed one sachet twice daily for eight weeks containing 2 g of a freeze-dried multi-species probiotic supplement (Ecologic^®^ AllergyCare, Winclove Probiotics B.V., Amsterdam, The Netherlands; probiotik^®^pur, nutrimmun GmbH, Munster, Germany). The supplement contained six bacterial strains, including *Bifidobacterium bifidum* W23, *Bifidobacterium lactis* W51, *Lactobacillus acidophilus* W55, *Lactobacillus casei* W56, *Lactobacillus salivarius* W57, and *Lactococcus lactis* W58 (total colony-forming units 1 × 10^9^/g), as well as vitamin B2 (35 mg/100 g), biotin (750 µg/100 g), maize starch and maltodextrins. As part of the study, patients provided a blood and a mucosal sample (nasal lavage with nasal brushing) for assessment of peripheral blood and nasal mucosal immune gene expression. From the main study a subset (*n* = 12) of samples were selected for analysis based on individual responses to the supplementation and clear classification as either responders or non-responders. Based on the criteria of Juniper et al., patients from the main study with a reduction in their mRQLQ score of 0.7 from pre- to post-supplement were classified as responders [7], while those with a reduction of less than 0.7 were classified as non-responders. Ethical approval was provided by the Griffith University Human Research Ethics Committee (Ref# 2015/564). 

Detailed methodology is included in the Supplementary Materials. Immune gene expression analysis of nasal cell lysate and isolated blood RNA samples was performed using the Nanostring nCounter PanCancer Immune Profiling Panel (NanoString Technologies, Seattle, WA, USA). Data were analysed with nSolver 4.0 (NanoString Technologies, Seattle, WA, USA) and TIGR Multi-Experiment Viewer [8]. The PanCancer Immune Profiling Panel allows for the assessment of 770 genes across 21 immune functional pathways and 24 immune cell subsets. The identification of immune-cell-specific genes allows for immune cell scoring [9] (supplementary methods). Group differences in immune gene expression, immune pathways, functional gene sets and immune cell scoring were compared using a Student’s *t*-test with data presented as mean ± standard deviation, with statistical significance accepted at *p* < 0.05 given the hypothesis-generating nature of this research. Clusters of functional protein–protein interactions were visualized using STRING 10.5 software [10], with a minimum confidence score of 0.90 to limit multiple comparisons of immune genes and false positives.

## 3. Results

### 3.1. Baseline Characteristics

**Figure 1:**
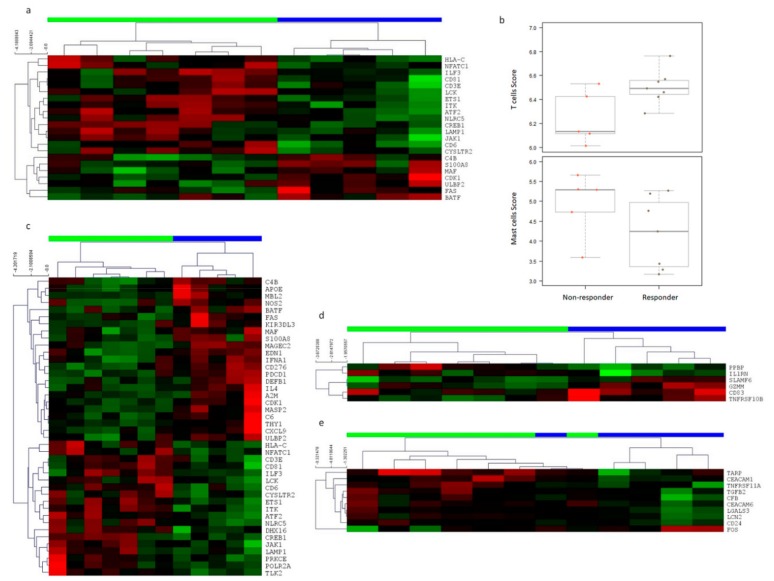
Immune gene expression in nasal lysate samples and whole blood at baseline and following supplementation in responders and non-responders. (**a**) Baseline whole-blood immune gene expression in responders vs. non-responders found 24 significantly differentially expressed genes (DEGs) between the groups. (**b**) Based on cell type scores, responders had a significantly higher abundance of T-cells and a trend for a lower abundance of mast cells in blood at baseline compared to responders (1: responders; 2: non-responders). (**c**) There were 42 genes significantly differentially expressed between responders and non-responders in nasal lysate samples at baseline. (**d**) Following supplementation, six immune genes in blood were significantly differentially expressed ≥1.5-fold between the groups. (**e**) There were 10 immune genes significantly different at ≥1.5-fold (*p* < 0.05) in nasal lavage/brushing samples between the groups. For Figure 1 (**a**,**c**,**d**,**e**) each row represents a gene (red: high expression; green: low expression) and each column a patient (green: responder; blue: non-responder). Rows are Z-score normalised.

Responders had a significantly higher baseline mRQLQ, sum of individual symptom scores and higher total white blood cell and lymphocyte count than non-responders (Table 1). There were 414 genes in blood at baseline expressed above the detection limit. The most significantly differentially expressed genes (DEGs) at baseline in blood with fold change (FC) ≥ 1.5 included *HLA-C* (FC 1.52; *p* = 0.0087) and *ETS1* (FC 1.51; *p* = 0.0018) (Supplementary Table S1)). Interestingly, two human leukocyte antigen genes displayed the largest fold-change difference between the groups, with *HLA-DQB1* expressing 3.7-fold (*p* = 0.11) and *HLA-DQA1* 1.76-fold (*p* = 0.13) higher in the responder group (Figure 1a). Immune cell scoring based on immune-cell-specific gene expression revealed a significantly higher abundance of T-cells and a trend for a lower abundance of mast cells in blood at baseline in responders (Figure 1b). 

There were 312 genes expressed above the detection limit (*n* = 312 genes) in the nasal lysate samples. A total of 64 genes at baseline were significantly differentially expressed in nasal lysate samples between the groups, with *IFIT2* expressed 8.9-fold (*p* = 0.004) and *CREB5* 14-fold (*p* = 0.005) higher in the responder group, and *IDO1* expressed 0.38-fold (*p* = 0.006) lower in the responder group (Figure 1c and Supplementary Table S2). Unsupervised hierarchical cluster analysis of the genes that were above the detection limit and significantly differentially expressed revealed two clusters that corresponded to responders and non-responders in blood and nasal lysate at baseline (Figure 1a,c). 

**Figure 2:**
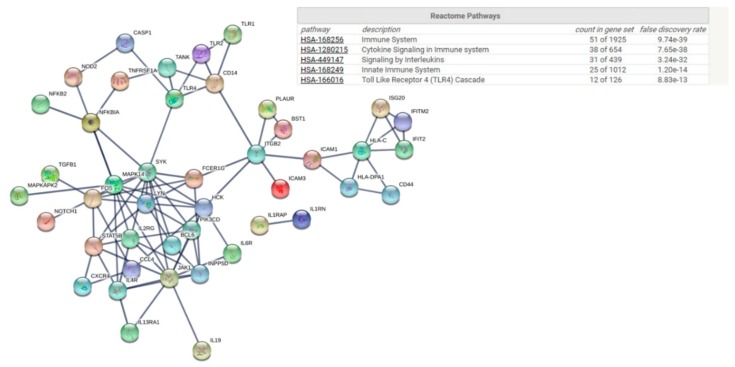
Protein–protein interaction (PPI) networks of immune DEGs between responders and non-responders in nasal lavage at baseline. Genes were clustered by pathway.

Functional protein–protein interaction analysis (STRING) identified DEGs with functional relationships in blood and the nasal mucosa. With only three DEGs with a >1.5-fold change (*p* < 0.05) in blood at baseline, no functional interaction networks were observed with STRING analysis. In the nasal mucosa (Figure 2), these included the immune system, cytokine signalling in the immune system and signalling by interleukins.

### 3.2. Effects of Supplementation

**Figure 3:**
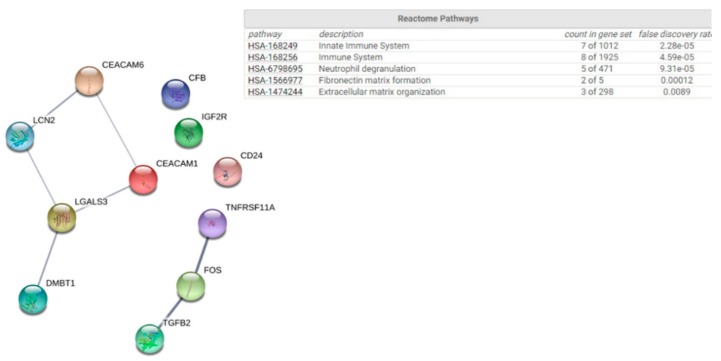
PPI of immune DEGs between responders and non-responders in nasal lavage following supplementation. Genes were clustered by pathway.

Patterns of differential immune gene expression in both whole blood and nasal lysate samples were also observed between responders and non-responders in response to probiotic supplementation. A clear pattern of immune gene expression separated the two groups in response to supplementation by selecting DEGs with a ≥1.5-fold change (*p* < 0.05) (Figure 1d and Supplementary Table S3). Furthermore, there was a significant 8% difference (*p* = 0.03) in Th-1 cell score abundance between the groups. This change in the abundance of Th-1 cells in the responder group is consistent with data from some allergy immunotherapy trials, in which decreases in Th-1 along with Th-2 cells in responders have been observed [11]. For the analysis of the nasal lysate samples and considering only the 446 genes expressed above limit of detection counts, there were 12 immune genes that had a ≥1.5-fold change (*p* < 0.05) difference between responders and non-responders in response to supplementation and that separated the groups (Figure 1e and Supplementary Table S4). We assessed the uncertainty in the hierarchical clusters for the effect of supplementation on immune gene expression in blood and nasal lysate with multiscale bootstrap sampling (*p* ≤ 0.001). Similar to baseline gene expression profiling, there were no groups of DEGs with functional relationships in blood following supplementation, but immune genes with a >1.5-fold (*p* < 0.05) difference in expression in nasal mucosal samples clustered into the immune system, followed by the innate immune system (Figure 3).

## 4. Discussion

While this was a proof-of-principle study, our data provide novel insights into the interaction between AR, inflammation and probiotic supplementation. Individuals classified as responders displayed clear immune gene signatures in blood and in nasal lysate samples at baseline and following probiotic supplementation. Given that responders had more severe symptoms both at baseline and following supplementation, these data suggest that individuals with a more severe allergic response may have an immune phenotype that may experience a greater benefit from supplementation. Interestingly, gene expression profiles in blood do not mirror those in mucosa. *ETS1*, which was significantly higher in blood at baseline in the responders, has previously been shown to be increased in allergen-challenged CD4+ cells from AR patients compared to controls [12]. *ETS1* regulates T-helper differentiation [13]. Interleukin-2-inducible T-cell kinase (ITK) is highly expressed in T-cells and has been implicated in a T-cell allergic rhinitis sub-type [14]. The higher expression of ITK and the higher abundance of T-cells in blood at baseline in the responder group, along with the observed reduction in the abundance of Th-1 cells in response to supplementation in responders, suggests that a distinct T-cell-mediated rhinitis sub-type may respond better to probiotic supplementation. Our observation of a significantly lower expression of *IDO1* in the responder group at baseline is consistent with conflicting reports of the role of indoleamine 2,3 dioxygenase (IDO) in allergy [15]. Interestingly, probiotic supplementation led to a significant 1.63-fold reduction in the expression of CD83 in blood in responders compared to non-responders. CD83+ dendritic cells have been shown to be higher in nasal mucosa biopsies in allergy [16]. DEGs at baseline and following supplementation could be enriched into specific functional groups via Gene Ontology (GO), the Kyoto Encyclopedia of Genes and Genomes (KEGG) and the Database for Annotation, Visualisation and Integrated Discovery (DAVID) pathways by STRING. At baseline DEGs between responders and non-responders were enriched into cytokine signalling, highlighting ongoing immune activity in disease. Following supplementation, DEGs clustered significantly into innate immune pathways. Key genes included *HLA-C*, *FCεR1G, TLR4,* and *MAPK14* at baseline. While our sample size is a limitation, the immune gene expression differences observed between responders and non-responders are consistent with our knowledge of the immunopathology of allergy [17]. It is necessary to extend these analyses into larger clinical trials to better understand whether sub-groups of allergy patients might benefit from daily probiotic supplementation. 

## Figures and Tables

**Table 1:** Baseline characteristics of responders and non-responders included in the immune gene expression analysis. * *p* < 0.05.

	Responders Mean ± SD	Non-Responders Mean ± SD
*n*	7	5
Gender (*n*)	3M/4F	3M/2F
Age (years)	43.71 ± 9.96	49.93 ± 15.53
Baseline mRQLQ score	3.72 ± 1.21	1.22 ± 1.05 *
Post-supplement mRQLQ score	1.30 ± 0.90	1.17 ± 0.90
Baseline sum of individual symptom score	22.43 ± 16.34	4.8 ± 4.55 *
Mono multi-allergy (*n*)	2/5	3/2
White cell count	7.41 ± 1.42	5.52 ± 1.02 *
Neutrophil	4.23 ± 1.2	3.26 ± 1.02
Lymphocyte	2.14 ± 0.24	1.54 ± 0.21 *
Monocyte	0.64 ± 0.25	0.52 ± 0.11
Eosinophil	0.36 ± 0.22	0.16 ± 0.07

M: male; F: female; mRQLQ: mini-rhinitis quality of life questionnaire.

## Supplementary Materials

The following are available online at https://www.mdpi.com/2073-4425/10/11/889/s1.
