# Supplementary material for: Digital Immune Gene Expression Profiling Discriminates Allergic Rhinitis Responders from Non-Responders to Probiotic Supplementation
Source: Genes (Basel). 2019 Nov 4;10(11):889. doi: 10.3390/genes10110889 (PMC6896104; doi:10.3390/genes10110889)
Supplement: Supplementary file 1 [file genes-10-00889-s001.pdf]

Supplementary material: detailed description of material and methods

Study design and participants

Full methodological details of the study design are published elsewhere [1]. Briefly, this phase II clinical study used a Simon Two-Stage design protocol to determine the response rate of allergic rhinitis (AR) sufferers to a multi-species probiotic supplement. Participants consumed a probiotic supplement twice daily for eight weeks and attended the allergy clinic at the beginning and end of intervention for assessment. The Simon Two-Stage design protocol was defined as p_1-p_0= 0.18 where p_0 (non-effective treatment) was nominated as a response in ≤15% of participants and p_1 (effective treatment) was as a response in least 33% of participants [2]. Response rates were calculated based on the proportion of participants reporting a clinically beneficial change in the validated mini Rhinoconjunctivitis Quality of Life Questionnaire (mRQLQ) [3] scores using a threshold of 0.7 as described in the work of Juniper et al. 2000.

Immune gene expression in blood and nasal lavage / brushing was assessed in a subset of participants; seven AR sufferers who were considered responders to the probiotic supplement and five AR sufferers who were considered non-responders based on the findings of the previously published trial [4].

Assessment of symptom severity

Symptom severity at baseline was assessed with an overall symptom survey, individual symptom questionnaire, and the mRQLQ, all of which were completed during the clinic visit. The overall symptom questionnaire assessed overall symptoms experienced in the previous 24 hours using an 11-point Likert scale (0 'No distress' to 10 'Unbearable distress'). The maximum overall symptom score was 10. The individual symptom questionnaire assessed specific symptoms (nasal itch, eye itch, sneezing, runny nose, post nasal drip, unrefreshed sleep and sinus pain) on the same 11-point likert scale. The scores for each symptom were summed to give a 'sum of individual symptoms' score with a possible maximum score of 70. The mRQLQ is a validated survey designed to measure the degree to which AR symptoms impact on the quality of life of an individual [3]. The survey consists of 15 questions separated into five sections; Activities, Practical problems, Nose Symptoms, Eye Symptoms, Other Symptoms. Each question is rated on a 7-point likert scale with the highest score indicating extremely troubled by symptoms and poorer quality of life.

Laboratory measures of immune parameters

Venous blood samples were collected from all participants at baseline. Analysis of white cell differential and specific IgE to Bermuda grass were completed by a local pathology provider (QML Pathology, Murarrie, Queensland, Australia). Erythrocyte sedimentation rate (ESR) over one hour was measured using fresh blood samples collected in sodium citrate tubes and using commercially available Vacuette ESR pipettes (Greiner Bio-One, Kremsmünster, Austria) as per the Westergren method [5]. Total serum IgE and IgG were measured in serum samples using commercially available enzyme-linked immunosorbent assay (ELISA) kits (eBioscience, San Diego, CA, USA).

Whole blood and nasal mucosa sampling

Venous blood samples were also collected into PAXgene RNA tubes (Pre-AnalytiX, Feldbachstrasse, Switzerland) and handled and stored according to the manufacturer's instructions. RNA was extracted from thawed PAXgene tubes with the Maxwell® RSC automated RNA extraction instrument using commercially available Maxwell® RSC miRNA Tissue Kit in accordance with the manufacturer's instructions. The quality and quantity of the extracted RNA was assessed spectrophotometrically with the NanoDrop 1000 UV-Vis spectrophotometer (ThermoScientific, Massachusettes, United States). RNA extracts were stored at -80 °C until analysis.

Nasal lavage samples were collected from participants with a modified nasal lavage procedure. Traditionally nasal lavage samples are collected by tilting the participants head backwards and instilling 5 ml of saline solution into each nasal cavity with fluid recovery after a predetermined dwell time. The method used in the current study was designed to sample a greater surface area of the nasal mucosa and sinuses. Using a nasal irrigation bottle (FLO Sinus Care, ENT technologies, Melbourne, Australia), participants administered 100 ml of PBS into each nostril and collected the fluid that was expelled from the free nostril into a sterile container. A volume of 20 ml of RMPI was then added to the lavage fluid to support the live cells until processing.

Nasal brushing was performed directly after the nasal lavage. Participants were advised to insert a brush (Piksters size 5, Erkstine oral care, Macksville, Australia) between the nasal septum and inferior turbinate and gently rotate the brush in circular and linear movements for 30 secs. The brush was then removed from the nasal cavity and suspended in a sterile container containing 3.5 ml RPMI. The procedure was then repeated in the other nostril with a fresh brush and placed into the same container following brushing. The nasal lavage and nasal brushing solutions were stored on ice until arrival at the laboratory.

Processing of nasal lavage and nasal brushing samples

The nasal lavage fluid was transferred into nuclease-free centrifuge tubes and centrifuged at 300 xg for 20 mins at 4 °C to concentrate cellular material. If substantial mucous was present, the lavage fluid was passed through a 100 µM filter (Corning, New York, United States) prior to centrifugation. Once centrifuged, the supernatant was removed and the resulting cell pellets resuspended in the residual volume and transferred into 1.5 ml nuclease-free microfuge tubes. In parallel, the nasal brushes were gently shaken in the RPMI solution to dislodge the nasal cells from the brush and discarded. The resulting cell brushing material was transferred into 1.5 ml nuclease free microfuge tubes. The microfuge tubes were spun at 14, 000 xg for 5 mins. The supernatant was removed and the cell pellets resuspended in residual volume. The resuspended cell pellets from the lavage and brushing samples were then pooled into a single 1.5 ml nuclease-free tube and centrifuged again at 14, 000 xg for 5 mins. The entire supernatant was removed and the resulting cell pellet was resuspended in commercially available lysis buffer (RLT, Qiagen, Hilden, Germany). The lysate was stored at -80 °C for downstream processing.

NanoString gene expression analysis

Immune gene expression analysis was undertaken using the NanoString nCounter analysis system (NanoString Technologies, Seattle, WA) using the commercially available nCounter PanCancer Immune Profiling panel kit together with the nCounter panel plus probe set of 30 immune genes (listed in Supplementary Table 5). The PanCancer Immune profiling panel contains n = 730 genes of key inflammatory pathways and n = 40 reference/housekeeping genes. The nCounter system directly detects and counts single-stranded nucleic acid via reporter probes affixed with flurophore barcodes and biotinylated capture-probes attached to microscopic beads. These are then affixed to lanes in cartridge and read in a digital scanner. Following the manufacturers protocol, 100 ng of total RNA extracted from whole blood or 5 µl of nasal cell lysate was hybridised with probes at 65 °C for 23 hours before being inserted into NanoString Prep Station were the target-probe complex was immobilised onto the analysis cartridge. Cartridges were read by the nCounter Digital Analyser for digital counting of molecular barcodes corresponding to each target at 280 fields of view.

Data approach

Statistical analysis for the baseline data (clinical chemistry, symptom severity, demographic data and white cell differential) was undertaken using GraphPad Prism V6.0 for Windows (GraphPad Software, La Jolla California USA). Data was log transformed for analysis and a Student's t-test used to compare responders and non-responders.

Gene expression data was analysed using the Advanced Analysis Module in the nSolver™ Analysis Software version 4.0 from NanoString Technologies (NanoString Technologies, WA, USA)

and TIGR Multi-Experiment Viewer (http://mev.tm4.org). The Advanced Analysis Module enables quality control (QC), normalisation, differential gene expression (DGE), Pathview Plots and immune cell profiling. Raw data was normalised by subtracting the mean plus one standard deviation of eight negative controls while technical variation was normalised through internal positive controls. Data was corrected for input volume via internal housekeeping genes using the geNorm algorithm. Genes that were expressed below 20 counts in more than 60% of samples were excluded from analysis. Differential gene expression was determined in blood and nasal lysate samples and significance between responders and non-responders determined using a t-test. Immune cell scores were determined using cell specific gene expression from The Cancer Genome Atlas (TCGA). Significance was accepted at $p < 0.05$ given the exploratory nature of the design. Clusters of functional protein-protein interactions were visualized using the STRING 10.5 software (https://string-db.org/), with a minimum confidence score of 0.90.

References

1. Watts, A.M., et al., *Probiotics and Allergic Rhinitis: A Simon Two-Stage Design to Determine Effectiveness.* The Journal of Alternative and Complementary Medicine, 2016. **22**(12): p. 1007-1012.
2. Watts, A.M., et al., Probiotics and Allergic Rhinitis: A Simon Two-Stage Design to Determine Effectiveness. J Altern Complement Med, 2016. **22**(12): p. 1007-1012.
3. Juniper, E.F., et al., Development and validation of the mini Rhinoconjunctivitis Quality of Life Questionnaire. Clin Exp Allergy, 2000. **30**(1): p. 132-40.
4. Watts, A.M., et al., A Specifically Designed Multispecies Probiotic Supplement Relieves Seasonal Allergic Rhinitis Symptoms. The Journal of Alternative and Complementary Medicine, 2018. **in-press**.
5. Von Boroviczeny, K.G., et al., Recommendation for Measurement of Erythrocyte Sedimentation Rate of Human Blood: International Committee For Standardization in Haematology. American Journal of Clinical Pathology, 1977. **68**(4): p. 505-507.

Supplementary Table S1: DEG in blood at baseline between responders and non-responders. There were 414 genes at baseline in blood expressed above background threshold. The table is order by fold change.

Gene	Log2 fold change	Lower confidence limit (log2)	Upper confidence limit (log2)	Linear fold change	Lower confidence limit (linear)	Upper confidence limit (linear)	*p*-value
HLA-DQA1	3.7	-0.436	7.84	13	0.739	229	0.11
HLA-DQB1	1.76	-0.331	3.85	3.38	0.795	14.4	0.13
BCL2	0.837	0.196	1.48	1.79	1.15	2.78	0.0284
IDO1	0.751	-0.108	1.61	1.68	0.928	3.05	0.118
LRRN3	0.626	-3.29	4.55	1.54	0.102	23.4	0.761
HLA-C	0.609	0.241	0.976	1.52	1.18	1.97	0.00876
ETS1	0.594	0.316	0.872	1.51	1.24	1.83	0.00186
NFATC1	0.571	0.222	0.92	1.49	1.17	1.89	0.0107
IL6ST	0.541	-0.0506	1.13	1.46	0.966	2.19	0.103
CCR7	0.525	-0.0335	1.08	1.44	0.977	2.12	0.0952
ITK	0.513	0.293	0.734	1.43	1.22	1.66	0.00105
LAIR2	0.504	0.0817	0.926	1.42	1.06	1.9	0.0414
C3AR1	0.497	-0.0765	1.07	1.41	0.948	2.1	0.12
MX1	0.499	-0.147	1.15	1.41	0.903	2.21	0.161
YTHDF2	0.45	-0.0817	0.982	1.37	0.945	1.97	0.128
NLRC5	0.451	0.184	0.718	1.37	1.14	1.65	0.00788
HRH4	0.454	-0.293	1.2	1.37	0.816	2.3	0.261
TXK	0.442	-3.68	4.56	1.36	0.0781	23.6	0.838
TCF7	0.431	0.0607	0.801	1.35	1.04	1.74	0.0457
SELL	0.423	-0.0472	0.893	1.34	0.968	1.86	0.108
CD6	0.396	0.129	0.663	1.32	1.09	1.58	0.0156
IGF1R	0.401	-0.0558	0.858	1.32	0.962	1.81	0.116
BTLA	0.406	0.00465	0.807	1.32	1	1.75	0.0755
IFNAR1	0.386	-4.86	5.63	1.31	0.0345	49.4	0.888
IFIT2	0.387	-0.413	1.19	1.31	0.751	2.28	0.365
IFI16	0.394	-0.044	0.831	1.31	0.97	1.78	0.108
IFIT1	0.375	-0.523	1.27	1.3	0.696	2.42	0.432
CMKLR1	0.376	-11.9	12.7	1.3	0.000256	6590	0.954
DDX58	0.378	-0.112	0.868	1.3	0.925	1.82	0.161
IFIH1	0.384	-0.0159	0.784	1.3	0.989	1.72	0.0892

THBS1	0.384	-2.72	3.48	1.3	0.152	11.2	0.814
CD3E	0.362	0.0791	0.646	1.29	1.06	1.56	0.031
CD5	0.366	-0.0563	0.788	1.29	0.962	1.73	0.12
MME	0.369	-0.203	0.941	1.29	0.869	1.92	0.235
CD81	0.371	0.114	0.628	1.29	1.08	1.55	0.0178
TLR10	0.354	-0.17	0.878	1.28	0.889	1.84	0.215
CD8A	0.356	-0.224	0.936	1.28	0.856	1.91	0.257
CD79A	0.358	-0.174	0.89	1.28	0.887	1.85	0.216
ITGA6	0.359	-0.218	0.935	1.28	0.86	1.91	0.251
OAS3	0.341	-0.164	0.845	1.27	0.893	1.8	0.215
EP300	0.343	0.0253	0.66	1.27	1.02	1.58	0.0604
CREB5	0.343	-0.252	0.938	1.27	0.84	1.92	0.285
LAMP1	0.344	0.0449	0.643	1.27	1.03	1.56	0.0479
CYBB	0.35	-0.138	0.837	1.27	0.909	1.79	0.19
CARD11	0.33	-0.0326	0.694	1.26	0.978	1.62	0.105
CREB1	0.338	0.0801	0.596	1.26	1.06	1.51	0.028
IGF2R	0.319	-0.212	0.85	1.25	0.863	1.8	0.266
TAPBP	0.32	0.0238	0.616	1.25	1.02	1.53	0.0602
CASP3	0.327	-0.0722	0.727	1.25	0.951	1.66	0.139
DOCK9	0.305	-0.0105	0.621	1.24	0.993	1.54	0.0873
FLT3LG	0.308	0.0449	0.572	1.24	1.03	1.49	0.0447
JAK3	0.312	-0.117	0.74	1.24	0.922	1.67	0.185
F13A1	0.315	-0.441	1.07	1.24	0.737	2.1	0.433
IL17RA	0.294	-0.0165	0.605	1.23	0.989	1.52	0.0932
CD28	0.302	-0.0848	0.688	1.23	0.943	1.61	0.157
CD9	0.304	-0.372	0.98	1.23	0.773	1.97	0.399
TNFRSF13C	0.283	-5.4	5.97	1.22	0.0236	62.7	0.924
ST6GAL1	0.271	0.0158	0.526	1.21	1.01	1.44	0.0641
PTGES2	0.271	-0.0338	0.575	1.21	0.977	1.49	0.115
IRF5	0.272	-0.0521	0.596	1.21	0.965	1.51	0.134
NFATC2	0.273	-0.00532	0.551	1.21	0.996	1.47	0.0835
IL18R1	0.276	-0.0819	0.633	1.21	0.945	1.55	0.162
NCR1	0.281	-0.338	0.899	1.21	0.791	1.86	0.394
TAB1	0.281	-15.1	15.6	1.21	2.90E-05	50900	0.972
CYLD	0.257	0.00766	0.507	1.2	1.01	1.42	0.071
CD7	0.258	-0.114	0.63	1.2	0.924	1.55	0.204

CD2	0.259	-5.8	6.32	1.2	0.018	79.7	0.935
LCK	0.261	0.0617	0.46	1.2	1.04	1.38	0.028
GZMM	0.264	-5.99	6.51	1.2	0.0158	91.3	0.936
CD4	0.266	-0.269	0.802	1.2	0.83	1.74	0.353
IL7R	0.269	-0.0269	0.564	1.2	0.982	1.48	0.105
MAP3K1	0.25	-0.118	0.619	1.19	0.921	1.54	0.213
ILF3	0.252	0.0309	0.473	1.19	1.02	1.39	0.0495
GTF3C1	0.255	-0.0713	0.581	1.19	0.952	1.5	0.157
CD24	0.234	-0.4	0.869	1.18	0.758	1.83	0.486
APP	0.236	-0.446	0.918	1.18	0.734	1.89	0.513
ITGA4	0.237	-0.0944	0.567	1.18	0.937	1.48	0.192
BMI1	0.237	-0.107	0.582	1.18	0.929	1.5	0.206
ENTPD1	0.238	-0.278	0.755	1.18	0.825	1.69	0.387
JAK1	0.244	-0.00502	0.493	1.18	0.997	1.41	0.0837
REL	0.22	-0.00725	0.448	1.17	0.995	1.36	0.087
CCR1	0.23	-0.435	0.896	1.17	0.74	1.86	0.513
IRF8	0.231	-0.229	0.692	1.17	0.853	1.62	0.349
STAT2	0.232	-0.0177	0.481	1.17	0.988	1.4	0.0987
ATF2	0.208	0.0471	0.37	1.16	1.03	1.29	0.0297
IL32	0.215	-0.078	0.507	1.16	0.947	1.42	0.181
PAFAH2	0.215	-6.66	7.09	1.16	0.00991	136	0.953
BCL6	0.218	-0.427	0.862	1.16	0.744	1.82	0.523
CXCR5	0.218	-5.08	5.51	1.16	0.0297	45.6	0.938
TAP2	0.196	-0.204	0.595	1.15	0.868	1.51	0.36
IKBKG	0.199	-0.218	0.616	1.15	0.859	1.53	0.372
ITGB1	0.201	-0.149	0.551	1.15	0.902	1.47	0.286
IL6R	0.201	-0.217	0.618	1.15	0.861	1.54	0.368
TRAF3	0.203	-0.169	0.576	1.15	0.889	1.49	0.309
CD36	0.205	-0.21	0.619	1.15	0.865	1.54	0.356
LCP1	0.184	-0.0669	0.435	1.14	0.955	1.35	0.181
CYFIP2	0.185	-0.0305	0.401	1.14	0.979	1.32	0.123
IL2RG	0.185	-0.0956	0.466	1.14	0.936	1.38	0.225
ADA	0.185	-0.215	0.586	1.14	0.861	1.5	0.386
MEF2C	0.187	-0.166	0.54	1.14	0.891	1.45	0.324
IFNAR2	0.189	-0.0244	0.402	1.14	0.983	1.32	0.113
CD96	0.191	-0.0524	0.434	1.14	0.964	1.35	0.155

CD74	0.172	-0.0328	0.376	1.13	0.978	1.3	0.131
TNFAIP3	0.172	-0.111	0.456	1.13	0.926	1.37	0.261
PTGDR2	0.174	-4.62	4.97	1.13	0.0407	31.2	0.945
ATG16L1	0.175	-0.127	0.478	1.13	0.915	1.39	0.283
LAMP2	0.175	-0.213	0.563	1.13	0.863	1.48	0.397
CXCL5	0.176	-1	1.36	1.13	0.499	2.56	0.776
IL2RB	0.179	-0.34	0.698	1.13	0.79	1.62	0.514
CD180	0.18	-0.39	0.751	1.13	0.763	1.68	0.55
IL4R	0.182	-0.252	0.615	1.13	0.84	1.53	0.43
PLA2G6	0.157	-5.87	6.18	1.12	0.0171	72.6	0.96
ZAP70	0.159	-0.0622	0.38	1.12	0.958	1.3	0.189
IKBKB	0.161	-0.199	0.521	1.12	0.871	1.43	0.401
CCR2	0.161	-0.297	0.619	1.12	0.814	1.54	0.506
HLA-DRB3	0.164	-0.243	0.571	1.12	0.845	1.49	0.449
CD46	0.165	-0.118	0.448	1.12	0.921	1.36	0.28
ANXA1	0.167	-0.167	0.502	1.12	0.89	1.42	0.35
ITGB2	0.168	-0.1	0.437	1.12	0.933	1.35	0.248
CD44	0.144	-0.103	0.392	1.11	0.931	1.31	0.28
GNLY	0.145	-0.907	1.2	1.11	0.533	2.29	0.793
EWSR1	0.146	-0.0885	0.38	1.11	0.941	1.3	0.251
NFATC3	0.149	-0.0833	0.382	1.11	0.944	1.3	0.237
SMAD3	0.149	-0.13	0.427	1.11	0.914	1.34	0.319
STAT1	0.15	-0.127	0.427	1.11	0.916	1.34	0.312
TNFRSF10B	0.152	-7.11	7.42	1.11	0.00722	171	0.968
ISG20	0.154	-0.249	0.557	1.11	0.841	1.47	0.472
IRF4	0.132	-6.39	6.65	1.1	0.0119	101	0.969
PRF1	0.137	-0.37	0.645	1.1	0.774	1.56	0.607
MS4A1	0.137	-0.394	0.669	1.1	0.761	1.59	0.623
CD247	0.138	-0.16	0.436	1.1	0.895	1.35	0.385
F2RL1	0.138	-3.93	4.21	1.1	0.0657	18.5	0.948
STAT3	0.141	-0.18	0.462	1.1	0.883	1.38	0.41
CD97	0.142	-0.149	0.433	1.1	0.902	1.35	0.36
IL1R1	0.142	-6.9	7.19	1.1	0.00835	146	0.969
ATG7	0.12	-0.168	0.407	1.09	0.89	1.33	0.434
BTK	0.12	-0.22	0.46	1.09	0.858	1.38	0.506
CD37	0.12	-0.228	0.467	1.09	0.854	1.38	0.515

RELB	0.122	-13.9	14.1	1.09	6.69E-05	17700	0.987
NR3C1	0.123	-0.0665	0.313	1.09	0.955	1.24	0.232
ITCH	0.123	-0.134	0.381	1.09	0.911	1.3	0.37
CD59	0.124	-0.304	0.553	1.09	0.81	1.47	0.582
CTLA4	0.125	-0.187	0.437	1.09	0.879	1.35	0.451
AKT3	0.126	-0.114	0.367	1.09	0.924	1.29	0.327
PIK3CD	0.128	-0.233	0.489	1.09	0.851	1.4	0.502
ITGA2B	0.128	-0.733	0.988	1.09	0.602	1.98	0.777
CD47	0.129	-0.0738	0.332	1.09	0.95	1.26	0.241
SELPLG	0.104	-0.212	0.421	1.08	0.863	1.34	0.532
TNFSF8	0.106	-0.219	0.431	1.08	0.859	1.35	0.536
MAP2K4	0.106	-0.353	0.564	1.08	0.783	1.48	0.661
CSF2RB	0.108	-0.314	0.53	1.08	0.804	1.44	0.627
ITGAL	0.109	-0.146	0.364	1.08	0.904	1.29	0.423
CD19	0.112	-0.563	0.787	1.08	0.677	1.73	0.751
NUP107	0.114	-0.133	0.36	1.08	0.912	1.28	0.387
PIK3CG	0.114	-0.175	0.403	1.08	0.886	1.32	0.458
CD86	0.115	-0.465	0.695	1.08	0.725	1.62	0.706
LTB	0.116	-0.167	0.4	1.08	0.891	1.32	0.439
JAK2	0.117	-0.136	0.371	1.08	0.91	1.29	0.386
PSEN1	0.0917	-0.269	0.452	1.07	0.83	1.37	0.629
TBX21	0.0937	-0.392	0.579	1.07	0.762	1.49	0.713
HLA-A	0.0964	-0.101	0.293	1.07	0.933	1.23	0.36
CDH1	0.097	-0.804	0.998	1.07	0.573	2	0.837
CR1	0.0992	-0.408	0.606	1.07	0.754	1.52	0.709
ICAM2	0.102	-0.176	0.38	1.07	0.885	1.3	0.489
CYSLTR1	0.103	-0.168	0.374	1.07	0.89	1.3	0.475
KLRG1	0.103	-0.41	0.615	1.07	0.753	1.53	0.703
ITGAM	0.0799	-0.276	0.436	1.06	0.826	1.35	0.67
RUNX3	0.0803	-0.287	0.447	1.06	0.82	1.36	0.677
LILRA1	0.0803	-0.364	0.525	1.06	0.777	1.44	0.73
SPN	0.0804	-0.249	0.41	1.06	0.841	1.33	0.643
ADORA2A	0.0822	-6.17	6.34	1.06	0.0138	80.9	0.98
IL1RAP	0.0829	-0.459	0.625	1.06	0.727	1.54	0.771
ITGAX	0.0841	-0.361	0.529	1.06	0.779	1.44	0.719
MAP3K7	0.0842	-0.0697	0.238	1.06	0.953	1.18	0.309

IRF7	0.0842	-0.349	0.517	1.06	0.785	1.43	0.711
LY9	0.0847	-0.167	0.336	1.06	0.891	1.26	0.525
FYN	0.0864	-0.136	0.309	1.06	0.91	1.24	0.464
KLRF1	0.0883	-0.699	0.875	1.06	0.616	1.83	0.83
PRKCD	0.0887	-0.241	0.418	1.06	0.846	1.34	0.609
TNFRSF1B	0.0888	-0.315	0.492	1.06	0.804	1.41	0.675
ALOX 5	0.0896	-0.239	0.419	1.06	0.847	1.34	0.605
CX3CR1	0.0648	-0.359	0.488	1.05	0.78	1.4	0.77
HRH2	0.066	-0.34	0.472	1.05	0.79	1.39	0.756
NLRP3	0.0664	-6.15	6.28	1.05	0.0141	77.9	0.984
C1QBP	0.0683	-0.181	0.317	1.05	0.882	1.25	0.602
MAP3K5	0.0723	-0.188	0.333	1.05	0.878	1.26	0.598
PECAM1	0.0727	-0.316	0.461	1.05	0.804	1.38	0.721
IRF2	0.0501	-0.218	0.318	1.04	0.86	1.25	0.722
CD3G	0.0535	-0.222	0.329	1.04	0.857	1.26	0.711
TOLLIP	0.0551	-0.155	0.265	1.04	0.898	1.2	0.618
MAVS	0.0568	-0.316	0.429	1.04	0.803	1.35	0.771
HLA-DOB	0.0577	-0.508	0.623	1.04	0.703	1.54	0.845
MR1	0.059	-13.2	13.3	1.04	0.00011	9900	0.993
MICB	0.0593	-0.199	0.317	1.04	0.871	1.25	0.662
NFKB2	0.0616	-0.36	0.483	1.04	0.779	1.4	0.78
SBNO2	0.0627	-0.312	0.437	1.04	0.806	1.35	0.749
HLA-DPB1	0.0632	-0.219	0.345	1.04	0.859	1.27	0.67
PTPRC	0.0368	-0.271	0.345	1.03	0.829	1.27	0.82
CD68	0.0369	-0.439	0.513	1.03	0.738	1.43	0.882
INPP5D	0.0373	-0.157	0.231	1.03	0.897	1.17	0.715
SYK	0.038	-0.252	0.328	1.03	0.84	1.26	0.803
CD1C	0.0422	-0.326	0.41	1.03	0.798	1.33	0.827
SIGIRR	0.0437	-0.199	0.287	1.03	0.871	1.22	0.732
CXCR3	0.044	-10	10.1	1.03	0.000959	1110	0.993
TXNIP	0.0455	-0.0704	0.161	1.03	0.952	1.12	0.459
TP53	0.0455	-0.301	0.392	1.03	0.812	1.31	0.802
ATG10	0.0476	-0.308	0.404	1.03	0.807	1.32	0.799
CTSW	0.0478	-0.439	0.535	1.03	0.738	1.45	0.851
CD22	0.0479	-0.399	0.495	1.03	0.758	1.41	0.838
TYK2	0.0482	-0.253	0.35	1.03	0.839	1.27	0.761

ICOS	0.0494	-0.271	0.37	1.03	0.829	1.29	0.769
IL1R2	0.0494	-0.485	0.584	1.03	0.715	1.5	0.86
TLR6	0.0229	-0.445	0.491	1.02	0.734	1.41	0.926
TLR2	0.0234	-0.531	0.578	1.02	0.692	1.49	0.936
ISG15	0.0241	-0.464	0.512	1.02	0.725	1.43	0.925
CEACAM1	0.0253	-6.26	6.31	1.02	0.013	79.5	0.994
CD79B	0.0293	-0.474	0.532	1.02	0.72	1.45	0.911
IL18RAP	0.035	-0.657	0.727	1.02	0.634	1.65	0.923
PTGS2	0.00721	-0.467	0.482	1.01	0.723	1.4	0.977
POU2F2	0.00839	-0.547	0.564	1.01	0.685	1.48	0.977
NOTCH1	0.00934	-0.331	0.349	1.01	0.795	1.27	0.958
CASP1	0.0113	-0.287	0.31	1.01	0.82	1.24	0.942
FCGR2A	0.0115	-0.486	0.509	1.01	0.714	1.42	0.965
SMAD2	0.0152	-0.211	0.242	1.01	0.864	1.18	0.898
TIGIT	0.0169	-8	8.03	1.01	0.00392	261	0.997
TAP1	0.0178	-0.265	0.301	1.01	0.832	1.23	0.904
PSMD7	0.0179	-0.182	0.217	1.01	0.882	1.16	0.864
KLRK1	0.0211	-0.456	0.498	1.01	0.729	1.41	0.933
CXCR4	0.00143	-0.182	0.185	1	0.881	1.14	0.988
HMGB1	0.00184	-0.121	0.125	1	0.919	1.09	0.977
TNFRSF1A	0.00299	-0.305	0.311	1	0.81	1.24	0.985
HCK	0.00417	-0.329	0.337	1	0.796	1.26	0.981
CD99	0.00448	-0.265	0.273	1	0.832	1.21	0.975
CCND3	-0.0024	-0.326	0.321	0.998	0.798	1.25	0.989
HLA-DPA1	-0.00485	-0.233	0.223	0.997	0.851	1.17	0.968
IFNGR1	-0.00392	-0.259	0.251	0.997	0.836	1.19	0.977
AMICA1	-0.00381	-0.285	0.278	0.997	0.821	1.21	0.979
MAPK14	-0.00769	-0.372	0.357	0.995	0.773	1.28	0.968
IL16	-0.00768	-0.167	0.151	0.995	0.891	1.11	0.926
ALCAM	-0.00837	-3.73	3.71	0.994	0.0754	13.1	0.997
STAT6	-0.0106	-0.403	0.382	0.993	0.756	1.3	0.959
RORA	-0.00982	-0.271	0.251	0.993	0.829	1.19	0.943
CCRL2	-0.00948	-5.8	5.78	0.993	0.018	54.8	0.998
HLA-DMB	-0.0122	-0.299	0.275	0.992	0.813	1.21	0.935
FPR2	-0.0111	-0.446	0.424	0.992	0.734	1.34	0.961
TICAM2	-0.0134	-8.72	8.7	0.991	0.00236	415	0.998

HLA-B	-0.0126	-0.283	0.258	0.991	0.822	1.2	0.929
MYD88	-0.0147	-0.255	0.225	0.99	0.838	1.17	0.907
IL10RA	-0.0161	-0.211	0.179	0.989	0.864	1.13	0.874
HAVCR2	-0.0192	-0.573	0.535	0.987	0.672	1.45	0.947
TLR8	-0.0188	-0.445	0.407	0.987	0.735	1.33	0.933
HLA-DMA	-0.0187	-0.249	0.212	0.987	0.842	1.16	0.877
TANK	-0.0212	-0.431	0.389	0.985	0.742	1.31	0.921
RUNX1	-0.0211	-0.359	0.316	0.985	0.78	1.25	0.905
LYN	-0.0233	-0.375	0.328	0.984	0.771	1.26	0.899
SLAMF7	-0.0226	-5.16	5.12	0.984	0.0279	34.7	0.993
STAT4	-0.0243	-0.398	0.349	0.983	0.759	1.27	0.901
CXCR2	-0.0262	-0.495	0.442	0.982	0.71	1.36	0.915
ANP32B	-0.0311	-0.252	0.19	0.979	0.839	1.14	0.788
CD40LG	-0.0357	-0.503	0.431	0.976	0.706	1.35	0.884
CD14	-0.035	-0.491	0.421	0.976	0.712	1.34	0.884
MAP2K2	-0.0371	-0.353	0.279	0.975	0.783	1.21	0.823
UBC	-0.0358	-0.228	0.156	0.975	0.854	1.11	0.722
TNFSF10	-0.0379	-0.348	0.272	0.974	0.786	1.21	0.816
PTGS1	-0.0374	-0.698	0.623	0.974	0.616	1.54	0.914
GPI	-0.0415	-0.371	0.288	0.972	0.773	1.22	0.81
IKBKE	-0.0408	-0.336	0.255	0.972	0.792	1.19	0.792
MAPK8	-0.0422	-8	7.92	0.971	0.0039	242	0.992
BCL10	-0.042	-0.28	0.196	0.971	0.823	1.15	0.737
HLA-E	-0.0439	-0.252	0.164	0.97	0.84	1.12	0.687
PNMA1	-0.0454	-0.403	0.312	0.969	0.756	1.24	0.809
NFKBIA	-0.0483	-0.263	0.166	0.967	0.833	1.12	0.668
STAT5B	-0.0514	-0.414	0.311	0.965	0.751	1.24	0.787
FCGR1A	-0.0541	-0.777	0.669	0.963	0.583	1.59	0.886
IL13RA1	-0.054	-0.267	0.159	0.963	0.831	1.12	0.63
CD53	-0.0579	-0.295	0.179	0.961	0.815	1.13	0.642
ITGA5	-0.0578	-0.276	0.16	0.961	0.826	1.12	0.614
CTSH	-0.0593	-0.583	0.465	0.96	0.667	1.38	0.829
TFEB	-0.0585	-0.52	0.403	0.96	0.698	1.32	0.809
IRF1	-0.0605	-0.285	0.164	0.959	0.821	1.12	0.609
LCN2	-0.0634	-0.777	0.65	0.957	0.583	1.57	0.865
TNFSF12	-0.0632	-0.321	0.195	0.957	0.8	1.14	0.641

TRAF2	-0.0655	-0.386	0.255	0.956	0.765	1.19	0.698
MIF	-0.0654	-0.5	0.369	0.956	0.707	1.29	0.774
IRAK4	-0.0648	-0.351	0.222	0.956	0.784	1.17	0.667
CXCR1	-0.0685	-0.539	0.402	0.954	0.688	1.32	0.781
CD84	-0.0681	-0.38	0.244	0.954	0.768	1.18	0.678
CD244	-0.0673	-0.424	0.29	0.954	0.745	1.22	0.72
CFP	-0.0743	-0.389	0.24	0.95	0.764	1.18	0.653
CTSS	-0.0742	-0.32	0.171	0.95	0.801	1.13	0.567
XCL2	-0.0779	-0.548	0.392	0.947	0.684	1.31	0.752
CASP8	-0.082	-0.363	0.199	0.945	0.778	1.15	0.58
BAX	-0.0817	-0.356	0.192	0.945	0.781	1.14	0.572
NCF4	-0.0834	-0.543	0.376	0.944	0.686	1.3	0.73
CD164	-0.0866	-0.246	0.073	0.942	0.843	1.05	0.313
CD63	-0.0864	-0.318	0.145	0.942	0.802	1.11	0.481
BCL2L1	-0.0863	-0.826	0.653	0.942	0.564	1.57	0.824
CCL5	-0.0913	-0.662	0.479	0.939	0.632	1.39	0.76
REPS1	-0.0966	-0.581	0.388	0.935	0.669	1.31	0.704
TLR1	-0.0989	-0.519	0.321	0.934	0.698	1.25	0.654
TGFB1	-0.0987	-0.386	0.189	0.934	0.765	1.14	0.516
IFI35	-0.1	-10	9.83	0.933	0.000956	910	0.985
CD40	-0.102	-0.533	0.329	0.932	0.691	1.26	0.652
MAPK3	-0.101	-0.457	0.255	0.932	0.729	1.19	0.59
FCGR3A	-0.101	-0.759	0.557	0.932	0.591	1.47	0.77
MAP2K1	-0.104	-0.377	0.169	0.931	0.77	1.12	0.473
LILRB3	-0.103	-0.58	0.373	0.931	0.669	1.3	0.68
CD3D	-0.104	-0.413	0.205	0.93	0.751	1.15	0.523
PLAUR	-0.104	-0.422	0.214	0.93	0.746	1.16	0.536
IRAK1	-0.108	-0.393	0.176	0.928	0.762	1.13	0.472
ITGAE	-0.108	-0.56	0.345	0.928	0.678	1.27	0.651
CXCL16	-0.111	-0.529	0.307	0.926	0.693	1.24	0.614
PF4	-0.113	-1.07	0.846	0.925	0.476	1.8	0.822
CD48	-0.113	-0.441	0.214	0.924	0.737	1.16	0.513
TNFRSF8	-0.115	-0.494	0.263	0.923	0.71	1.2	0.564
CD1D	-0.117	-0.503	0.269	0.922	0.706	1.21	0.566
TLR4	-0.117	-0.609	0.375	0.922	0.656	1.3	0.652
FCGR2B	-0.119	-0.642	0.403	0.921	0.641	1.32	0.664

FUT7	-0.125	-0.59	0.341	0.917	0.664	1.27	0.611
PYCARD	-0.124	-0.543	0.294	0.917	0.687	1.23	0.573
CD27	-0.127	-0.393	0.14	0.916	0.761	1.1	0.373
IFITM1	-0.127	-0.554	0.3	0.916	0.681	1.23	0.573
BST2	-0.128	-0.399	0.144	0.915	0.758	1.1	0.378
MAPKAPK2	-0.138	-0.392	0.116	0.909	0.762	1.08	0.313
SH2D1A	-0.137	-0.637	0.362	0.909	0.643	1.29	0.602
PSMB9	-0.139	-0.337	0.0595	0.908	0.791	1.04	0.2
PSMB10	-0.141	-0.371	0.0893	0.907	0.773	1.06	0.258
IL12RB1	-0.141	-0.455	0.173	0.907	0.729	1.13	0.402
TNFRSF14	-0.144	-0.346	0.0576	0.905	0.786	1.04	0.191
ATG5	-0.146	-0.47	0.178	0.904	0.722	1.13	0.399
FOS	-0.146	-0.676	0.385	0.904	0.626	1.31	0.602
CAMP	-0.145	-1.02	0.732	0.904	0.492	1.66	0.752
PSMB8	-0.15	-0.338	0.0386	0.901	0.791	1.03	0.15
CHUK	-0.154	-0.457	0.148	0.899	0.729	1.11	0.341
MAPK1	-0.154	-0.549	0.241	0.899	0.684	1.18	0.464
NOD2	-0.155	-0.792	0.481	0.898	0.578	1.4	0.642
CSF3R	-0.159	-0.535	0.218	0.896	0.69	1.16	0.428
KLRD1	-0.159	-0.812	0.494	0.896	0.57	1.41	0.643
TNFSF13	-0.158	-5.68	5.37	0.896	0.0194	41.3	0.957
TNFRSF10C	-0.166	-0.683	0.351	0.892	0.623	1.28	0.544
GZMH	-0.17	-0.899	0.56	0.889	0.536	1.47	0.658
ICAM3	-0.176	-0.483	0.131	0.885	0.716	1.1	0.288
BST1	-0.178	-0.546	0.19	0.884	0.685	1.14	0.366
TNFSF13B	-0.178	-0.637	0.281	0.884	0.643	1.22	0.466
RIPK2	-0.179	-0.378	0.0202	0.883	0.769	1.01	0.109
TRAF6	-0.183	-0.481	0.114	0.881	0.717	1.08	0.254
ATF1	-0.188	-0.457	0.0817	0.878	0.729	1.06	0.202
PPBP	-0.187	-1.02	0.643	0.878	0.494	1.56	0.668
RPS6	-0.189	-0.609	0.231	0.877	0.655	1.17	0.398
IL1B	-0.189	-0.648	0.27	0.877	0.638	1.21	0.439
ICAM1	-0.191	-0.789	0.406	0.876	0.579	1.33	0.544
SH2D1B	-0.195	-0.93	0.54	0.874	0.525	1.45	0.614
PSMB7	-0.199	-0.389	-0.00893	0.871	0.763	0.994	0.0673
TBK1	-0.203	-0.491	0.0847	0.869	0.711	1.06	0.197

GZMA	-0.202	-0.624	0.22	0.869	0.649	1.16	0.371
HLA-DRA	-0.214	-0.389	-0.0387	0.862	0.764	0.974	0.0377
CD58	-0.214	-0.504	0.0768	0.862	0.705	1.05	0.18
IL2RA	-0.216	-0.449	0.0166	0.861	0.732	1.01	0.0987
LTF	-0.218	-1.19	0.754	0.86	0.438	1.69	0.669
FCER1G	-0.223	-0.699	0.254	0.857	0.616	1.19	0.382
LILRB2	-0.224	-0.597	0.149	0.856	0.661	1.11	0.266
GZMB	-0.224	-0.953	0.505	0.856	0.516	1.42	0.56
TSC22D3	-0.234	-0.748	0.281	0.851	0.595	1.21	0.394
TREM1	-0.238	-0.753	0.276	0.848	0.594	1.21	0.385
CD33	-0.242	-0.657	0.173	0.845	0.634	1.13	0.279
MEFV	-0.253	-0.648	0.142	0.839	0.638	1.1	0.238
SPA17	-0.258	-5.64	5.12	0.837	0.0201	34.9	0.927
HLA-G	-0.267	-0.722	0.189	0.831	0.606	1.14	0.278
IL1RN	-0.27	-0.899	0.359	0.829	0.536	1.28	0.42
DUSP6	-0.273	-0.584	0.0387	0.828	0.667	1.03	0.117
IFITM2	-0.275	-0.705	0.155	0.827	0.613	1.11	0.239
ABCB1	-0.274	-0.566	0.0189	0.827	0.675	1.01	0.0999
TFRC	-0.276	-1.04	0.488	0.826	0.487	1.4	0.495
LILRB1	-0.282	-0.547	-0.0166	0.823	0.684	0.989	0.0639
MAP4K2	-0.281	-0.544	-0.0178	0.823	0.686	0.988	0.0628
SLC11A1	-0.286	-0.834	0.263	0.82	0.561	1.2	0.332
CLEC4A	-0.291	-0.519	-0.0626	0.817	0.698	0.958	0.0316
CD160	-0.296	-4.32	3.73	0.815	0.0499	13.3	0.889
TARP	-0.297	-0.789	0.195	0.814	0.579	1.14	0.264
TNF	-0.305	-0.641	0.0316	0.81	0.641	1.02	0.106
MAF	-0.308	-0.654	0.037	0.808	0.636	1.03	0.111
ECSIT	-0.314	-0.678	0.0509	0.805	0.625	1.04	0.123
LY86	-0.317	-0.655	0.0199	0.802	0.635	1.01	0.0949
KLRC2	-0.32	-1.27	0.626	0.801	0.416	1.54	0.523
CD8B	-0.322	-7.45	6.81	0.8	0.00571	112	0.931
CCL4	-0.324	-0.925	0.278	0.799	0.527	1.21	0.316
PIN1	-0.333	-0.598	-0.0682	0.794	0.661	0.954	0.0334
IL24	-0.338	-4.09	3.41	0.791	0.0588	10.6	0.864
GZMK	-0.342	-0.92	0.236	0.789	0.528	1.18	0.273
CEBPB	-0.344	-0.732	0.0429	0.788	0.602	1.03	0.112

CFD	-0.347	-1.42	0.728	0.786	0.373	1.66	0.541
CCR3	-0.356	-0.937	0.225	0.781	0.522	1.17	0.257
CCL3	-0.359	-1.42	0.701	0.78	0.374	1.63	0.522
TNFSF14	-0.369	-0.669	-0.0701	0.774	0.629	0.953	0.0361
KLRB1	-0.371	-0.802	0.0595	0.773	0.574	1.04	0.122
LGALS3	-0.373	-1	0.256	0.772	0.499	1.19	0.272
IL15RA	-0.386	-1.36	0.591	0.765	0.389	1.51	0.457
LTBR	-0.389	-0.783	0.00466	0.764	0.581	1	0.0815
CKLF	-0.431	-0.838	-0.0238	0.742	0.559	0.984	0.0648
S100A12	-0.439	-1.07	0.194	0.738	0.476	1.14	0.204
CLEC7A	-0.443	-0.9	0.0148	0.736	0.536	1.01	0.0871
KLRC1	-0.448	-1.2	0.309	0.733	0.434	1.24	0.273
BATF	-0.457	-0.76	-0.155	0.728	0.591	0.898	0.0159
LILRA5	-0.463	-0.974	0.0479	0.725	0.509	1.03	0.106
CXCL1	-0.475	-1.13	0.179	0.72	0.457	1.13	0.185
TNFSF4	-0.485	-0.963	-0.00833	0.714	0.513	0.994	0.0741
LY96	-0.504	-1.03	0.0223	0.705	0.49	1.02	0.09
S100A8	-0.542	-0.962	-0.122	0.687	0.513	0.919	0.03
LTB4R	-0.541	-6.49	5.41	0.687	0.0111	42.4	0.863
THBD	-0.631	-1.53	0.264	0.646	0.347	1.2	0.197
FCER1A	-0.672	-1.34	-0.00617	0.627	0.395	0.996	0.0761
C4B	-0.78	-4.67	3.11	0.583	0.0394	8.62	0.703
IL8	-0.87	-1.83	0.0889	0.547	0.281	1.06	0.106
SLPI	-1.09	-1.83	-0.343	0.471	0.282	0.788	0.0168

Supplementary Table S2. Differential gene expression in nasal lysate at baseline between responders and non-responders.

Genes	Log2 fold change	Lower confidence limit (log2)	Upper confidence limit (log2)	Linear fold change	Lower confidence limit (linear)	Upper confidence limit (linear)	*p*-value
IFIT2	3.16	1.49	4.83	8.94	2.82	28.4	0.00399
CREB5	3.83	1.75	5.91	14.2	3.37	59.9	0.00474
IDO1	-1.39	-2.19	-0.599	0.381	0.219	0.66	0.00635
BST1	1.91	0.787	3.03	3.75	1.73	8.15	0.00751
IL1RAP	2.18	0.825	3.54	4.54	1.77	11.6	0.0103
CXCR4	3.44	1.3	5.59	10.9	2.45	48	0.0105
PTGS2	3.52	1.29	5.76	11.5	2.45	54.1	0.0114
IL6R	2.65	0.951	4.35	6.27	1.93	20.3	0.0121
IL17RA	2.22	0.792	3.65	4.67	1.73	12.6	0.0123
PIK3CD	2.85	1.01	4.7	7.22	2.01	26	0.0128
PLAUR	3.23	1.12	5.34	9.39	2.17	40.6	0.0134
PNMA1	-0.641	-1.07	-0.214	0.641	0.477	0.862	0.0147
ICAM3	2.74	0.914	4.57	6.68	1.88	23.7	0.0148
IL1RN	2.15	0.719	3.59	4.45	1.65	12	0.0148
ALOX 5	2.8	0.909	4.68	6.95	1.88	25.7	0.0157
TGFB1	2.27	0.73	3.8	4.81	1.66	13.9	0.0161
INPP5D	1.87	0.599	3.14	3.65	1.51	8.79	0.0162
IGF2R	1.49	0.471	2.52	2.82	1.39	5.73	0.0169
CCL4	3.17	0.987	5.36	9.01	1.98	41	0.0174
EGR2	2.39	0.736	4.05	5.26	1.67	16.6	0.0179
CKLF	1.85	0.557	3.14	3.6	1.47	8.81	0.0186
FOS	1.43	0.43	2.44	2.7	1.35	5.42	0.0189
MAPK14	1.03	0.306	1.76	2.05	1.24	3.39	0.0193
NOTCH1	1.67	0.493	2.85	3.18	1.41	7.19	0.0194
ITGB2	2.9	0.848	4.95	7.47	1.8	31	0.0198
CCL3L1	3.08	0.883	5.28	8.47	1.84	38.9	0.0206
HCK	3.09	0.884	5.3	8.53	1.85	39.5	0.0207
IL16	2.46	0.688	4.23	5.49	1.61	18.7	0.0215
CD44	1.15	0.317	1.98	2.22	1.25	3.96	0.0221
IFITM2	2.76	0.749	4.77	6.76	1.68	27.2	0.0227
TLR4	2.79	0.753	4.83	6.92	1.69	28.4	0.0229
TLR2	2.43	0.603	4.26	5.39	1.52	19.1	0.0262

AMICA1	2.88	0.676	5.09	7.38	1.6	34.1	0.0283
TNFRSF1A	1.24	0.29	2.18	2.36	1.22	4.54	0.0284
CD83	2.39	0.551	4.22	5.23	1.46	18.6	0.0289
CD97	1.88	0.432	3.33	3.68	1.35	10	0.0291
LTB	2.53	0.575	4.49	5.79	1.49	22.5	0.0296
NOD2	1.89	0.423	3.37	3.72	1.34	10.3	0.0302
LCP1	2.73	0.606	4.86	6.65	1.52	29	0.0304
TLR1	2.18	0.477	3.87	4.52	1.39	14.7	0.0309
STAT5B	1.69	0.368	3.02	3.24	1.29	8.12	0.0312
NFKB2	1.47	0.318	2.62	2.77	1.25	6.15	0.0314
MAPKAPK2	0.806	0.174	1.44	1.75	1.13	2.71	0.0314
IL13RA1	0.768	0.163	1.37	1.7	1.12	2.59	0.0322
SYK	1.24	0.255	2.22	2.36	1.19	4.66	0.0332
MAP3K7	0.41	0.0831	0.737	1.33	1.06	1.67	0.0338
CD14	2.52	0.502	4.54	5.73	1.42	23.2	0.0344
ICAM1	2.29	0.444	4.14	4.9	1.36	17.6	0.0354
CLEC4A	2.32	0.448	4.19	4.98	1.36	18.2	0.0355
IL2RG	2.2	0.406	4	4.61	1.33	16	0.0371
TANK	1.4	0.255	2.54	2.63	1.19	5.8	0.0374
LYN	1.39	0.247	2.53	2.62	1.19	5.79	0.0384
IL19	-2.07	-3.78	-0.368	0.237	0.0727	0.775	0.0384
FCER1G	2.56	0.437	4.67	5.88	1.35	25.5	0.0397
BCL6	1.39	0.22	2.56	2.62	1.17	5.89	0.0421
HLA-DPA1	1.94	0.295	3.59	3.84	1.23	12	0.0434
CCRL2	2	0.298	3.7	3.99	1.23	13	0.044
NFKBIA	1.65	0.239	3.07	3.15	1.18	8.4	0.0449
CASP1	1.78	0.249	3.3	3.42	1.19	9.85	0.0458
ISG20	1.27	0.168	2.36	2.41	1.12	5.15	0.0473
HLA-C	0.869	0.114	1.62	1.83	1.08	3.08	0.0478
IL4R	1.2	0.156	2.25	2.3	1.11	4.75	0.048
JAK1	0.669	0.0851	1.25	1.59	1.06	2.38	0.0485
IKBKG	0.489	0.0601	0.917	1.4	1.04	1.89	0.0495
NOS2A	-0.97	-1.83	-0.111	0.51	0.281	0.926	0.0514
IRAK2	0.908	0.101	1.71	1.88	1.07	3.28	0.0519
TBK1	0.347	0.0377	0.656	1.27	1.03	1.58	0.0525
CASP8	1.25	0.135	2.37	2.38	1.1	5.16	0.0527

STAT3	0.735	0.0775	1.39	1.66	1.06	2.62	0.0532
TFRC	0.52	0.0546	0.986	1.43	1.04	1.98	0.0533
YTHDF2	-0.703	-1.33	-0.0736	0.614	0.397	0.95	0.0534
TXNIP	0.86	0.0697	1.65	1.82	1.05	3.14	0.0587
ITGA5	2.07	0.165	3.98	4.2	1.12	15.7	0.059
IFNGR1	1.07	0.0678	2.07	2.1	1.05	4.2	0.0629
IL10RA	2.05	0.118	3.99	4.15	1.09	15.8	0.0642
DUSP6	1.04	0.0597	2.02	2.06	1.04	4.05	0.0643
TNFAIP3	1.29	0.0721	2.5	2.44	1.05	5.67	0.0646
LY96	2.09	0.109	4.07	4.25	1.08	16.8	0.0655
HLA-DPB1	2.39	0.0586	4.72	5.23	1.04	26.3	0.0723
TIRAP	-0.431	-0.853	-0.0101	0.742	0.554	0.993	0.0725
TYK2	0.57	0.0123	1.13	1.48	1.01	2.18	0.073
LAMP2	0.776	0.015	1.54	1.71	1.01	2.9	0.0736
NOS2	-0.877	-1.74	-0.00907	0.545	0.298	0.994	0.0758
CD59	-0.348	-0.694	-0.00147	0.786	0.618	0.999	0.0774
CD274	0.909	-0.00138	1.82	1.88	0.999	3.53	0.0788
MAP2K1	0.517	-0.0019	1.04	1.43	0.999	2.05	0.0794
IL1R1	0.903	-0.00799	1.81	1.87	0.994	3.52	0.0807
S100A8	1.89	-0.0247	3.8	3.71	0.983	14	0.0818
HLA-E	0.948	-0.0128	1.91	1.93	0.991	3.76	0.0819
CREB1	0.329	-0.00686	0.664	1.26	0.995	1.58	0.0838
PSEN1	0.409	-0.00905	0.827	1.33	0.994	1.77	0.0841
C2	-1.13	-2.29	0.0298	0.457	0.205	1.02	0.0853
CD46	0.637	-0.0204	1.29	1.56	0.986	2.45	0.0867
CFD	1.96	-0.0636	3.99	3.9	0.957	15.9	0.0868
IRAK4	0.557	-0.0205	1.13	1.47	0.986	2.19	0.088
CCL28	-1.08	-2.21	0.045	0.472	0.216	1.03	0.0892
KIT	-1.17	-2.4	0.0659	0.446	0.19	1.05	0.0933
CYLD	0.726	-0.043	1.49	1.65	0.971	2.82	0.094
EP300	0.556	-0.0369	1.15	1.47	0.975	2.22	0.0959
CTSS	1.14	-0.0857	2.36	2.2	0.942	5.14	0.0983
OAS3	-0.692	-1.44	0.0601	0.619	0.368	1.04	0.102
CDKN1A	0.747	-0.0772	1.57	1.68	0.948	2.97	0.106
ATG7	0.751	-0.0976	1.6	1.68	0.935	3.03	0.113
TOLLIP	0.317	-0.042	0.677	1.25	0.971	1.6	0.114

THBD	1.39	-0.186	2.96	2.62	0.879	7.8	0.115
HLA-DRA	1.29	-0.176	2.75	2.44	0.885	6.74	0.115
PRKCD	0.734	-0.102	1.57	1.66	0.932	2.97	0.116
SBNO2	0.987	-0.148	2.12	1.98	0.902	4.36	0.119
TNFRSF14	0.557	-0.0853	1.2	1.47	0.943	2.3	0.12
RUNX3	0.843	-0.134	1.82	1.79	0.911	3.53	0.122
IRF7	0.803	-0.147	1.75	1.75	0.903	3.37	0.129
IFNAR2	0.559	-0.103	1.22	1.47	0.931	2.33	0.129
CYFIP2	1.14	-0.212	2.48	2.2	0.863	5.59	0.13
IL1R2	2.8	-0.534	6.14	6.97	0.691	70.4	0.131
CD68	0.805	-0.154	1.76	1.75	0.899	3.39	0.131
TFEB	0.998	-0.198	2.19	2	0.872	4.58	0.133
IFI27	-2.47	-5.42	0.493	0.181	0.0233	1.41	0.133
NUP107	-0.704	-1.55	0.141	0.614	0.341	1.1	0.134
JAK2	0.435	-0.096	0.966	1.35	0.936	1.95	0.139
CD58	0.732	-0.168	1.63	1.66	0.89	3.1	0.142
NFKB1	0.626	-0.144	1.4	1.54	0.905	2.63	0.142
HLA-DQA1	3.3	-0.767	7.38	9.88	0.587	166	0.143
MAP3K1	0.301	-0.0706	0.673	1.23	0.952	1.59	0.143
TSC22D3	0.981	-0.234	2.2	1.97	0.85	4.59	0.145
RELA	0.302	-0.0726	0.676	1.23	0.951	1.6	0.145
PSMB10	-0.229	-0.514	0.0558	0.853	0.7	1.04	0.146
GTF3C1	-0.343	-0.772	0.0861	0.788	0.586	1.06	0.148
IRF1	0.748	-0.197	1.69	1.68	0.872	3.23	0.152
IFIH1	0.667	-0.182	1.52	1.59	0.881	2.86	0.155
MAPK1	0.441	-0.121	1	1.36	0.92	2	0.155
BAX	0.25	-0.0713	0.571	1.19	0.952	1.49	0.158
HLA-DRB3	0.991	-0.287	2.27	1.99	0.819	4.82	0.16
CFB	-0.824	-1.89	0.241	0.565	0.27	1.18	0.161
LRP1	-0.778	-1.79	0.231	0.583	0.29	1.17	0.162
LIF	1.28	-0.418	2.98	2.43	0.749	7.9	0.17
CD276	-0.379	-0.882	0.123	0.769	0.543	1.09	0.17
THBS1	1.45	-0.489	3.39	2.74	0.713	10.5	0.173
CXCL1	1.1	-0.383	2.59	2.15	0.767	6.02	0.176
ATG5	-0.374	-0.878	0.129	0.771	0.544	1.09	0.176
RUNX1	-0.446	-1.05	0.154	0.734	0.484	1.11	0.176

STAT6	0.483	-0.17	1.14	1.4	0.889	2.2	0.178
CSF2RB	2.6	-0.954	6.15	6.06	0.516	71.1	0.182
PLAU	1.11	-0.413	2.63	2.15	0.751	6.17	0.184
MYD88	0.677	-0.258	1.61	1.6	0.836	3.06	0.186
IFITM1	1.66	-0.633	3.95	3.15	0.645	15.4	0.187
CXCL16	0.61	-0.247	1.47	1.53	0.842	2.77	0.193
SAA1	-0.709	-1.71	0.293	0.612	0.305	1.23	0.196
SELL	2.26	-0.97	5.49	4.79	0.511	44.9	0.2
ABL1	-0.506	-1.24	0.225	0.704	0.424	1.17	0.205
BID	0.838	-0.381	2.06	1.79	0.768	4.16	0.208
UBC	0.343	-0.156	0.841	1.27	0.898	1.79	0.208
ALCAM	-0.397	-0.974	0.181	0.76	0.509	1.13	0.208
LCN2	-0.569	-1.41	0.272	0.674	0.377	1.21	0.214
PSMB7	-0.337	-0.838	0.164	0.792	0.56	1.12	0.216
IKBKE	-0.734	-1.84	0.37	0.601	0.28	1.29	0.222
CXCL3	1.07	-0.56	2.71	2.11	0.678	6.54	0.227
RIPK2	0.842	-0.445	2.13	1.79	0.734	4.37	0.229
CHUK	0.249	-0.135	0.632	1.19	0.911	1.55	0.232
CD24	-0.469	-1.19	0.256	0.723	0.437	1.19	0.234
HLA-B	0.462	-0.259	1.18	1.38	0.836	2.27	0.238
VEGFA	0.628	-0.369	1.62	1.55	0.774	3.08	0.245
ITGB1	-0.237	-0.617	0.142	0.848	0.652	1.1	0.248
TRAF3	0.276	-0.173	0.725	1.21	0.887	1.65	0.255
VEGFC	-0.874	-2.29	0.544	0.546	0.204	1.46	0.255
NR3C1	0.481	-0.301	1.26	1.4	0.812	2.4	0.256
NLRC5	0.385	-0.253	1.02	1.31	0.839	2.03	0.264
TP53	-1.28	-3.42	0.857	0.411	0.0932	1.81	0.267
IKBKB	-0.284	-0.759	0.192	0.822	0.591	1.14	0.269
CASP3	-0.345	-0.927	0.236	0.787	0.526	1.18	0.271
MUC1	-0.378	-1.02	0.26	0.769	0.494	1.2	0.273
PSEN2	-0.813	-2.19	0.561	0.569	0.22	1.48	0.273
FCGR2A	2.38	-1.65	6.41	5.2	0.319	84.9	0.274
CLEC7A	2.06	-1.46	5.58	4.17	0.362	47.9	0.279
REPS1	-0.16	-0.435	0.116	0.895	0.74	1.08	0.282
ITGAX	2.04	-1.52	5.6	4.11	0.348	48.6	0.288
MAP2K4	0.281	-0.212	0.775	1.22	0.863	1.71	0.29

IGF1R	0.234	-0.178	0.645	1.18	0.884	1.56	0.291
ANXA1	0.413	-0.316	1.14	1.33	0.803	2.21	0.293
FOXJ1	-1.18	-3.26	0.902	0.442	0.105	1.87	0.293
SIGIRR	-0.388	-1.09	0.31	0.764	0.471	1.24	0.301
HLA-DMB	0.583	-0.474	1.64	1.5	0.72	3.12	0.305
OSM	2.06	-1.71	5.82	4.16	0.305	56.7	0.31
SPA17	-1.07	-3.04	0.895	0.475	0.122	1.86	0.31
IL8	1.8	-1.56	5.16	3.48	0.339	35.7	0.319
RORC	-0.607	-1.74	0.53	0.657	0.298	1.44	0.32
PECAM1	1.9	-1.67	5.46	3.72	0.314	44.1	0.322
ATF2	0.215	-0.191	0.621	1.16	0.876	1.54	0.324
MAP3K5	0.364	-0.325	1.05	1.29	0.798	2.07	0.325
CXCR2	2.07	-1.88	6.02	4.19	0.271	64.7	0.329
EGR1	0.667	-0.607	1.94	1.59	0.657	3.84	0.329
BATF	0.418	-0.389	1.23	1.34	0.764	2.34	0.334
ITGA2	0.318	-0.304	0.94	1.25	0.81	1.92	0.34
DDX58	0.386	-0.377	1.15	1.31	0.77	2.22	0.345
IRAK1	0.26	-0.254	0.775	1.2	0.838	1.71	0.345
RRAD	-0.912	-2.72	0.895	0.531	0.152	1.86	0.346
PSMB8	-0.147	-0.441	0.147	0.903	0.737	1.11	0.35
MERTK	0.527	-0.552	1.61	1.44	0.682	3.05	0.361
CXCL6	0.818	-0.869	2.51	1.76	0.548	5.68	0.364
S100A7	2.09	-2.23	6.41	4.26	0.214	84.8	0.365
EPCAM	-0.615	-1.9	0.667	0.653	0.269	1.59	0.369
TNFRSF10C	1.96	-2.15	6.07	3.9	0.226	67.2	0.371
ITGA6	-0.727	-2.25	0.794	0.604	0.21	1.73	0.371
IL1B	2.09	-2.31	6.49	4.26	0.202	90	0.373
FPR2	1.96	-2.2	6.11	3.88	0.218	69.1	0.378
PYCARD	0.233	-0.264	0.729	1.18	0.833	1.66	0.38
BCL10	0.29	-0.334	0.915	1.22	0.793	1.89	0.384
HLA-DMA	0.381	-0.45	1.21	1.3	0.732	2.32	0.39
MX1	-0.353	-1.12	0.416	0.783	0.46	1.33	0.39
IL33	-0.596	-1.9	0.711	0.662	0.268	1.64	0.393
IRF2	0.384	-0.464	1.23	1.31	0.725	2.35	0.395
CD74	0.47	-0.576	1.52	1.39	0.671	2.86	0.399
TNFSF10	-0.172	-0.554	0.211	0.888	0.681	1.16	0.399

REL	0.339	-0.439	1.12	1.27	0.738	2.17	0.413
IL6ST	0.166	-0.215	0.547	1.12	0.861	1.46	0.414
PSMD7	-0.148	-0.497	0.201	0.903	0.708	1.15	0.426
CD63	-0.155	-0.522	0.212	0.898	0.696	1.16	0.426
ATF1	-0.26	-0.874	0.354	0.835	0.545	1.28	0.426
PTPRC	1.57	-2.15	5.29	2.97	0.225	39.1	0.428
CEACAM1	0.558	-0.775	1.89	1.47	0.584	3.71	0.431
TREM1	1.94	-2.76	6.64	3.84	0.147	100	0.437
TNFRSF10B	0.243	-0.352	0.837	1.18	0.783	1.79	0.442
IL15RA	0.363	-0.541	1.27	1.29	0.687	2.4	0.45
IRF3	-0.749	-2.63	1.13	0.595	0.161	2.19	0.454
BST2	-0.502	-1.79	0.782	0.706	0.29	1.72	0.461
CD53	1.41	-2.21	5.03	2.65	0.216	32.6	0.464
ITCH	0.28	-0.444	1	1.21	0.735	2	0.466
PIN1	-0.154	-0.554	0.247	0.899	0.681	1.19	0.469
BCL2L1	0.112	-0.193	0.417	1.08	0.875	1.34	0.487
ANP32B	-0.16	-0.594	0.274	0.895	0.663	1.21	0.487
MAVS	-0.186	-0.696	0.325	0.879	0.617	1.25	0.492
SPINK5	0.321	-0.563	1.2	1.25	0.677	2.31	0.493
STAT1	0.25	-0.455	0.955	1.19	0.729	1.94	0.503
IFI16	0.166	-0.312	0.644	1.12	0.806	1.56	0.511
LGALS3	-0.214	-0.835	0.408	0.862	0.56	1.33	0.516
CSF3R	1.52	-2.96	6	2.86	0.128	63.8	0.522
PSMB9	0.312	-0.613	1.24	1.24	0.654	2.36	0.523
CEACAM6	-0.33	-1.33	0.671	0.796	0.398	1.59	0.533
MAPK3	-0.155	-0.632	0.322	0.898	0.645	1.25	0.539
TNFRSF18	-0.477	-1.96	1	0.718	0.258	2	0.541
CDH1	-0.267	-1.1	0.563	0.831	0.468	1.48	0.542
LTBR	0.156	-0.35	0.663	1.11	0.785	1.58	0.558
NFATC1	-0.3	-1.28	0.676	0.812	0.413	1.6	0.56
APP	-0.184	-0.787	0.42	0.88	0.579	1.34	0.564
HMGB1	-0.31	-1.33	0.713	0.807	0.397	1.64	0.565
TAP1	0.247	-0.572	1.07	1.19	0.672	2.1	0.567
CTSH	-0.175	-0.761	0.411	0.886	0.59	1.33	0.572
CCL3	0.986	-2.38	4.35	1.98	0.193	20.4	0.578
C3	-0.441	-1.95	1.06	0.736	0.26	2.09	0.578

MST1R	-0.276	-1.22	0.67	0.826	0.429	1.59	0.58
PAFAH2	-0.355	-1.58	0.873	0.782	0.334	1.83	0.584
HLA-A	0.212	-0.528	0.953	1.16	0.693	1.94	0.587
CD55	0.366	-0.953	1.69	1.29	0.517	3.22	0.598
ARG2	-0.313	-1.44	0.814	0.805	0.369	1.76	0.598
CD9	0.284	-0.745	1.31	1.22	0.597	2.48	0.6
IFNAR1	0.189	-0.52	0.898	1.14	0.697	1.86	0.613
RORA	-0.22	-1.05	0.605	0.859	0.484	1.52	0.613
IL18	-0.326	-1.55	0.898	0.798	0.342	1.86	0.613
LAMP1	-0.137	-0.652	0.378	0.909	0.636	1.3	0.614
EWSR1	0.0598	-0.174	0.294	1.04	0.886	1.23	0.627
CD47	0.097	-0.284	0.478	1.07	0.821	1.39	0.628
PTGES2	-0.123	-0.61	0.363	0.918	0.655	1.29	0.631
TGFB2	-0.404	-2.05	1.24	0.756	0.241	2.37	0.641
TPSAB1	-0.536	-2.74	1.67	0.69	0.149	3.18	0.644
TAPBP	0.141	-0.461	0.744	1.1	0.726	1.67	0.656
C1QBP	-0.218	-1.17	0.729	0.859	0.446	1.66	0.661
ILF3	-0.0464	-0.253	0.16	0.968	0.839	1.12	0.668
BMI1	-0.295	-1.65	1.06	0.815	0.319	2.08	0.679
ST6GAL1	-0.238	-1.35	0.871	0.848	0.393	1.83	0.683
SMAD3	-0.132	-0.767	0.502	0.912	0.588	1.42	0.691
NFATC3	-0.175	-1.03	0.677	0.886	0.491	1.6	0.696
TNFSF13	-0.147	-0.903	0.609	0.903	0.535	1.53	0.711
BLNK	0.115	-0.504	0.735	1.08	0.705	1.66	0.723
ATG16L1	0.128	-0.592	0.847	1.09	0.664	1.8	0.735
TRAF2	0.102	-0.485	0.69	1.07	0.714	1.61	0.74
DUSP4	-0.159	-1.12	0.801	0.895	0.46	1.74	0.752
DOCK9	-0.143	-1.01	0.724	0.906	0.497	1.65	0.753
MAP2K2	0.0564	-0.29	0.403	1.04	0.818	1.32	0.756
PLA2G6	-0.176	-1.26	0.907	0.885	0.418	1.87	0.757
IFI35	-0.0684	-0.492	0.355	0.954	0.711	1.28	0.758
MAPK8	0.103	-0.534	0.739	1.07	0.69	1.67	0.759
JUN	-0.139	-1.05	0.773	0.908	0.483	1.71	0.771
CD40	-0.154	-1.28	0.974	0.899	0.411	1.96	0.795
MAF	0.153	-1	1.31	1.11	0.5	2.47	0.801
CCND3	0.0847	-0.557	0.726	1.06	0.68	1.65	0.801

ETS1	0.121	-0.885	1.13	1.09	0.541	2.18	0.819
SERPINB2	0.181	-1.43	1.79	1.13	0.372	3.46	0.83
RPS6	-0.066	-0.674	0.542	0.955	0.627	1.46	0.836
TAP2	0.0561	-0.501	0.613	1.04	0.706	1.53	0.848
F2RL1	0.0565	-0.524	0.637	1.04	0.696	1.55	0.852
GPI	0.074	-0.691	0.839	1.05	0.619	1.79	0.854
SH2D1B	0.114	-1.23	1.45	1.08	0.428	2.74	0.871
CD36	-0.119	-1.66	1.42	0.921	0.316	2.68	0.882
LTF	0.0995	-1.27	1.47	1.07	0.414	2.77	0.89
CD81	0.0543	-0.695	0.804	1.04	0.618	1.75	0.89
TRAF6	0.0313	-0.409	0.471	1.02	0.753	1.39	0.892
CEBPB	0.308	-4.24	4.86	1.24	0.053	28.9	0.897
MFGE8	0.0496	-0.773	0.873	1.03	0.585	1.83	0.908
TJP1	-0.0372	-0.765	0.691	0.975	0.588	1.61	0.922
MIF	0.038	-0.713	0.789	1.03	0.61	1.73	0.923
CD99	-0.0412	-0.973	0.89	0.972	0.51	1.85	0.933
SMAD2	-0.0626	-1.48	1.35	0.958	0.359	2.55	0.933
CD164	0.0968	-2.23	2.43	1.07	0.212	5.38	0.937
CTSL	0.0501	-1.17	1.27	1.04	0.445	2.41	0.937
SCGB1A1	-0.0633	-1.77	1.64	0.957	0.294	3.12	0.943
ISG15	0.0544	-1.49	1.6	1.04	0.356	3.03	0.946
TICAM1	0.0307	-0.988	1.05	1.02	0.504	2.07	0.954
STAT2	-0.00924	-0.373	0.355	0.994	0.772	1.28	0.961
SLPI	-0.0118	-0.841	0.818	0.992	0.558	1.76	0.978
ITGB4	0.0125	-0.897	0.922	1.01	0.537	1.89	0.979
TAB1	-0.00924	-0.794	0.776	0.994	0.577	1.71	0.982
CXCL2	-0.0277	-3.54	3.49	0.981	0.0859	11.2	0.988
CLDN1	-0.00364	-1.15	1.15	0.997	0.449	2.21	0.995

Supplementary Table S3. Differentially expressed genes between responders and non-responders in blood following supplementation.

Genes	Log2 fold change	Lower confidence limit (log2)	Upper confidence limit (log2)	Linear fold change	Lower confidence limit (linear)	Upper confidence limit (linear)	*p*-value
IL24	0.98	-0.07	2.04	1.98	0.95	4.10	0.0649
HLA-DRB4	0.96	-0.13	2.04	1.94	0.92	4.11	0.0786
PPBP	0.89	0.25	1.53	1.85	1.19	2.88	0.0103
TFRC	0.89	0.06	1.71	1.85	1.05	3.27	0.0367
IL1RN	0.80	0.14	1.45	1.74	1.10	2.73	0.0211
IL15RA	0.75	-0.14	1.64	1.68	0.91	3.11	0.0931
RELB	0.73	-0.18	1.64	1.66	0.88	3.12	0.1082
CXCL5	0.67	-0.26	1.61	1.59	0.83	3.05	0.1449
PF4	0.67	-0.06	1.39	1.59	0.96	2.63	0.0675
IFIT1	0.63	0.07	1.19	1.55	1.05	2.29	0.0298
FEZ1	0.63	-0.16	1.42	1.55	0.90	2.67	0.1066
CD9	0.62	-0.33	1.57	1.54	0.79	2.98	0.1824
TNFRSF17	0.62	-0.50	1.73	1.53	0.71	3.33	0.2539
JAM3	0.61	0.02	1.20	1.52	1.01	2.29	0.0452
LY96	0.58	0.14	1.01	1.49	1.11	2.01	0.0129
CD8B	0.56	0.07	1.05	1.47	1.05	2.06	0.0290
FCER1A	0.52	-0.27	1.30	1.43	0.83	2.47	0.1789
IL18	0.51	-0.49	1.51	1.42	0.71	2.85	0.2895
IFITM1	0.51	-0.07	1.08	1.42	0.95	2.12	0.0800
LILRB1	0.49	0.15	0.84	1.41	1.11	1.79	0.0089
PIN1	0.49	-0.12	1.10	1.41	0.92	2.14	0.1040
LILRA1	0.48	-0.20	1.17	1.40	0.87	2.25	0.1506
IL2RA	0.48	-0.24	1.21	1.40	0.84	2.31	0.1744
PTGDR2	0.47	-0.52	1.46	1.39	0.70	2.74	0.3207
FCGR2B	0.46	-0.38	1.29	1.37	0.77	2.45	0.2585
LTA	0.45	-0.03	0.94	1.37	0.98	1.91	0.0648
S100A8	0.45	-0.03	0.93	1.37	0.98	1.91	0.0654
LGALS3	0.45	-0.13	1.03	1.36	0.91	2.04	0.1202
BCL2L1	0.42	-0.28	1.13	1.34	0.82	2.19	0.2181
ICAM1	0.42	-0.30	1.14	1.34	0.81	2.21	0.2322
RPS6	0.42	-0.24	1.07	1.33	0.85	2.10	0.1915
RNASE3	0.41	-0.81	1.64	1.33	0.57	3.11	0.4753

RIPK2	0.41	-0.17	0.98	1.33	0.89	1.97	0.1489
CKLF	0.41	-0.07	0.88	1.32	0.95	1.84	0.0890
IFITM2	0.40	-0.14	0.93	1.32	0.91	1.91	0.1306
TNFSF4	0.39	-0.05	0.83	1.31	0.97	1.77	0.0767
S100A12	0.39	-0.21	0.98	1.31	0.87	1.97	0.1806
BATF	0.37	-0.27	1.01	1.30	0.83	2.02	0.2280
CDH1	0.37	-0.47	1.20	1.29	0.72	2.30	0.3598
MAPK3	0.36	-0.09	0.81	1.28	0.94	1.75	0.1110
BST1	0.36	-0.05	0.77	1.28	0.96	1.70	0.0824
MAGEA3	0.34	-0.29	0.96	1.26	0.82	1.95	0.2630
REPS1	0.34	-0.16	0.84	1.26	0.89	1.79	0.1668
TANK	0.33	-0.08	0.75	1.26	0.95	1.68	0.1057
THBD	0.33	-0.21	0.87	1.25	0.86	1.82	0.2137
IL8	0.33	-0.29	0.94	1.25	0.82	1.92	0.2706
PSMB8	0.32	-0.09	0.74	1.25	0.94	1.67	0.1170
C4B	0.32	-0.37	1.01	1.25	0.77	2.02	0.3308
TLR5	0.32	-0.31	0.95	1.25	0.81	1.93	0.2894
APP	0.32	-0.34	0.98	1.25	0.79	1.97	0.3155
CAMP	0.32	-0.47	1.10	1.25	0.72	2.14	0.3965
CD48	0.32	-0.16	0.79	1.25	0.90	1.73	0.1735
MAP2K1	0.31	-0.14	0.75	1.24	0.91	1.68	0.1588
CD19	0.31	-0.26	0.87	1.24	0.83	1.83	0.2641
ECSIT	0.31	-0.33	0.94	1.24	0.80	1.91	0.3136
MAPK1	0.30	-0.12	0.72	1.23	0.92	1.65	0.1457
PSMB7	0.30	-0.05	0.65	1.23	0.96	1.57	0.0900
CTLA4	0.30	-0.26	0.85	1.23	0.83	1.81	0.2717
MIF	0.29	-0.25	0.83	1.22	0.84	1.78	0.2688
IRAK1	0.29	-0.38	0.95	1.22	0.77	1.94	0.3674
HMGB1	0.29	-0.15	0.72	1.22	0.90	1.65	0.1745
SSX1	0.28	-0.52	1.09	1.22	0.70	2.12	0.4599
CCR7	0.28	-0.43	0.99	1.22	0.74	1.99	0.4053
IL1B	0.28	-0.12	0.68	1.21	0.92	1.60	0.1525
TNFSF12	0.28	-0.18	0.74	1.21	0.88	1.67	0.2127
ABCB1	0.28	-0.27	0.83	1.21	0.83	1.78	0.2940
C1QBP	0.28	-0.37	0.93	1.21	0.77	1.90	0.3739
IFIT2	0.28	-0.21	0.77	1.21	0.86	1.70	0.2449

CFD	0.27	-0.37	0.92	1.21	0.77	1.89	0.3734
TNFRSF14	0.27	-0.23	0.78	1.21	0.85	1.71	0.2613
HLA-DPA1	0.26	-0.06	0.58	1.20	0.96	1.50	0.1044
CD2	0.26	-0.51	1.02	1.19	0.70	2.02	0.4798
CD59	0.25	-0.18	0.68	1.19	0.88	1.60	0.2294
CEBPB	0.25	-0.23	0.72	1.19	0.86	1.65	0.2768
CXCL1	0.25	-0.37	0.87	1.19	0.77	1.82	0.4035
ADORA2A	0.25	-0.50	0.99	1.19	0.71	1.99	0.4846
LY86	0.25	-0.29	0.78	1.19	0.82	1.72	0.3411
BMI1	0.24	-0.12	0.61	1.18	0.92	1.53	0.1751
SLPI	0.24	-0.56	1.04	1.18	0.68	2.06	0.5270
ISG15	0.24	-0.26	0.74	1.18	0.84	1.67	0.3171
IRAK4	0.24	-0.23	0.71	1.18	0.85	1.64	0.2894
TNFSF13B	0.24	-0.18	0.66	1.18	0.88	1.58	0.2424
FCGR3A	0.24	-0.22	0.70	1.18	0.86	1.62	0.2852
BST2	0.23	-0.20	0.66	1.17	0.87	1.59	0.2678
CD36	0.23	-0.24	0.69	1.17	0.85	1.61	0.3073
ISG20	0.23	-0.24	0.69	1.17	0.85	1.61	0.3116
MAPK8	0.22	-0.38	0.82	1.16	0.77	1.77	0.4433
CCR1	0.22	-0.20	0.64	1.16	0.87	1.56	0.2809
ANP32B	0.22	-0.21	0.65	1.16	0.86	1.57	0.2959
PSMB10	0.21	-0.13	0.55	1.16	0.92	1.46	0.1970
CD27	0.21	-0.21	0.62	1.15	0.86	1.54	0.3070
KLRB1	0.19	-0.41	0.79	1.14	0.75	1.73	0.5095
CASP1	0.19	-0.16	0.54	1.14	0.89	1.45	0.2692
CD68	0.18	-0.45	0.81	1.13	0.73	1.75	0.5467
CCL5	0.17	-0.25	0.59	1.13	0.84	1.51	0.3954
CD3D	0.17	-0.31	0.65	1.13	0.81	1.57	0.4561
IL13RA1	0.17	-0.31	0.65	1.12	0.81	1.56	0.4533
CD53	0.17	-0.22	0.55	1.12	0.86	1.46	0.3599
ICAM3	0.17	-0.27	0.61	1.12	0.83	1.53	0.4255
FPR2	0.17	-0.27	0.60	1.12	0.83	1.52	0.4226
TPSAB1	0.16	-0.83	1.14	1.11	0.56	2.21	0.7371
HLA-G	0.15	-0.28	0.58	1.11	0.83	1.50	0.4551
SBNO2	0.15	-0.41	0.71	1.11	0.75	1.64	0.5769
TBK1	0.15	-0.29	0.59	1.11	0.82	1.51	0.4791

TRAF3	0.14	-0.42	0.71	1.10	0.75	1.64	0.5942
IL18RAP	0.14	-0.29	0.58	1.10	0.82	1.49	0.4962
CCR3	0.14	-0.44	0.71	1.10	0.74	1.64	0.6091
TTK	0.13	-0.76	1.03	1.10	0.59	2.05	0.7505
CD1C	0.13	-0.40	0.67	1.10	0.76	1.59	0.5962
CEACAM1	0.13	-0.85	1.11	1.10	0.56	2.16	0.7737
SMAD2	0.13	-0.22	0.48	1.10	0.86	1.40	0.4307
SELPLG	0.13	-0.29	0.55	1.10	0.82	1.46	0.5060
TSC22D3	0.13	-0.37	0.63	1.10	0.78	1.55	0.5777
TNF	0.13	-0.26	0.52	1.09	0.84	1.43	0.4795
TNFRSF10C	0.13	-0.37	0.63	1.09	0.77	1.55	0.5854
TNFSF14	0.13	-0.25	0.51	1.09	0.84	1.42	0.4805
HLA-DRA	0.13	-0.26	0.52	1.09	0.83	1.43	0.4887
IL23A	0.13	-0.31	0.56	1.09	0.81	1.47	0.5394
NUP107	0.13	-0.24	0.49	1.09	0.85	1.40	0.4666
ALCAM	0.12	-0.84	1.09	1.09	0.56	2.12	0.7829
PSEN1	0.12	-0.38	0.62	1.09	0.77	1.54	0.6023
BAX	0.12	-0.39	0.63	1.09	0.76	1.55	0.6139
FCER1G	0.12	-0.30	0.53	1.08	0.81	1.44	0.5544
KLRC2	0.12	-0.57	0.80	1.08	0.67	1.74	0.7211
SPA17	0.11	-0.78	1.00	1.08	0.58	2.01	0.7939
CTSH	0.11	-0.37	0.58	1.08	0.77	1.50	0.6355
CCL3L1	0.11	-0.55	0.77	1.08	0.68	1.70	0.7326
CSF3R	0.11	-0.28	0.50	1.08	0.82	1.41	0.5645
TLR2	0.11	-0.40	0.61	1.08	0.76	1.53	0.6565
HLA-DPB1	0.11	-0.29	0.50	1.08	0.82	1.41	0.5698
CD3G	0.10	-0.35	0.56	1.08	0.79	1.47	0.6261
PLAUR	0.10	-0.33	0.53	1.07	0.80	1.44	0.6155
LTBR	0.10	-0.34	0.55	1.07	0.79	1.46	0.6295
TLR4	0.10	-0.36	0.56	1.07	0.78	1.48	0.6450
IL4R	0.10	-0.29	0.49	1.07	0.82	1.41	0.5914
PSMB9	0.10	-0.26	0.46	1.07	0.83	1.37	0.5721
CXCR1	0.10	-0.41	0.60	1.07	0.75	1.51	0.6878
CTSS	0.09	-0.25	0.44	1.07	0.84	1.36	0.5684
MAF	0.09	-0.45	0.63	1.06	0.73	1.55	0.7263
NFKBIA	0.09	-0.28	0.45	1.06	0.82	1.37	0.6205

MAP2K4	0.08	-0.43	0.60	1.06	0.74	1.52	0.7299
TLR1	0.08	-0.35	0.51	1.06	0.79	1.43	0.6797
CXCR4	0.08	-0.36	0.53	1.06	0.78	1.44	0.6905
PYCARD	0.08	-0.54	0.70	1.06	0.69	1.63	0.7813
F13A1	0.08	-0.62	0.78	1.06	0.65	1.72	0.8083
ATF1	0.08	-0.36	0.52	1.06	0.78	1.44	0.7033
CLEC7A	0.07	-0.27	0.41	1.05	0.83	1.33	0.6606
ANXA1	0.07	-0.29	0.43	1.05	0.82	1.35	0.6783
LTB4R	0.07	-0.58	0.72	1.05	0.67	1.65	0.8201
TLR6	0.07	-0.31	0.45	1.05	0.81	1.37	0.6992
IL1RAP	0.07	-0.56	0.70	1.05	0.68	1.62	0.8180
ITGAE	0.06	-0.69	0.82	1.05	0.62	1.77	0.8554
AMICA1	0.06	-0.28	0.41	1.05	0.83	1.32	0.6895
SIGIRR	0.06	-0.52	0.64	1.04	0.70	1.56	0.8176
FUT7	0.06	-0.44	0.56	1.04	0.74	1.48	0.7907
PIK3CD	0.06	-0.43	0.56	1.04	0.74	1.47	0.7918
MAPKAPK2	0.06	-0.40	0.52	1.04	0.76	1.44	0.7794
ITGA4	0.06	-0.29	0.41	1.04	0.82	1.33	0.7129
CCL4	0.06	-0.47	0.58	1.04	0.72	1.50	0.8123
CXCL16	0.06	-0.46	0.57	1.04	0.73	1.48	0.8141
TREM1	0.06	-0.39	0.50	1.04	0.76	1.42	0.7933
JAK2	0.05	-0.34	0.45	1.04	0.79	1.37	0.7760
VEGFA	0.05	-0.59	0.69	1.04	0.66	1.62	0.8659
TNFSF10	0.05	-0.27	0.37	1.03	0.83	1.29	0.7413
POU2F2	0.05	-0.50	0.60	1.03	0.71	1.52	0.8490
PSMD7	0.05	-0.50	0.60	1.03	0.71	1.51	0.8484
TP53	0.05	-0.38	0.47	1.03	0.77	1.39	0.8159
ATG10	0.05	-0.57	0.66	1.03	0.67	1.58	0.8730
LYN	0.04	-0.52	0.61	1.03	0.70	1.53	0.8666
CD58	0.04	-0.33	0.42	1.03	0.79	1.34	0.8020
SYK	0.04	-0.45	0.53	1.03	0.73	1.45	0.8590
CD46	0.04	-0.31	0.38	1.03	0.81	1.30	0.8210
GZMK	0.04	-0.37	0.45	1.02	0.77	1.36	0.8546
CYSLTR1	0.04	-0.49	0.56	1.02	0.71	1.47	0.8858
STAT5B	0.04	-0.41	0.48	1.02	0.75	1.40	0.8675
TAB1	0.04	-0.50	0.57	1.02	0.71	1.49	0.8895

CHUK	0.03	-0.44	0.51	1.02	0.74	1.42	0.8794
BTK	0.03	-0.46	0.52	1.02	0.73	1.43	0.8977
IRF1	0.03	-0.43	0.48	1.02	0.74	1.40	0.8931
LTB	0.03	-0.29	0.35	1.02	0.82	1.27	0.8516
PTGS1	0.03	-0.42	0.47	1.02	0.75	1.38	0.8969
CLEC4A	0.02	-0.41	0.46	1.02	0.75	1.37	0.9098
IFI27	0.02	-1.11	1.16	1.02	0.46	2.23	0.9661
IL1R2	0.02	-0.58	0.62	1.02	0.67	1.54	0.9364
NCF4	0.02	-0.46	0.50	1.01	0.73	1.41	0.9370
UBC	0.02	-0.34	0.38	1.01	0.79	1.30	0.9202
CD33	0.02	-0.56	0.60	1.01	0.68	1.51	0.9523
CTSW	0.01	-0.52	0.55	1.01	0.70	1.46	0.9522
MX1	0.01	-0.36	0.39	1.01	0.78	1.31	0.9410
CD40	0.01	-0.56	0.58	1.01	0.68	1.49	0.9657
STAT4	0.01	-0.46	0.48	1.01	0.72	1.40	0.9645
ARG1	0.01	-0.74	0.75	1.01	0.60	1.69	0.9789
CD1D	0.01	-0.39	0.41	1.01	0.76	1.32	0.9609
HLA-DMA	0.01	-0.51	0.53	1.01	0.70	1.44	0.9709
ITGA5	0.01	-0.38	0.40	1.00	0.77	1.32	0.9699
CYFIP2	0.01	-0.35	0.36	1.00	0.79	1.28	0.9735
CD63	0.00	-0.41	0.41	1.00	0.75	1.33	0.9884
GZMA	0.00	-0.49	0.50	1.00	0.71	1.41	0.9922
CD160	0.00	-0.46	0.47	1.00	0.73	1.38	0.9929
SLC11A1	0.00	-0.45	0.44	1.00	0.73	1.35	0.9817
GPI	-0.01	-0.50	0.48	1.00	0.71	1.40	0.9805
BCL10	-0.01	-0.44	0.43	1.00	0.74	1.34	0.9770
ITCH	-0.01	-0.44	0.43	1.00	0.74	1.34	0.9749
MAP4K2	-0.01	-0.49	0.48	1.00	0.71	1.39	0.9775
IRF7	-0.01	-0.64	0.63	1.00	0.64	1.55	0.9816
TRAF2	-0.01	-0.63	0.61	0.99	0.64	1.53	0.9714
ATG5	-0.01	-0.51	0.49	0.99	0.70	1.41	0.9637
TOLLIP	-0.01	-0.38	0.36	0.99	0.77	1.28	0.9450
PNMA1	-0.01	-0.47	0.44	0.99	0.72	1.36	0.9451
FCGR2A	-0.02	-0.48	0.45	0.99	0.72	1.36	0.9358
KLRD1	-0.02	-0.52	0.49	0.99	0.70	1.40	0.9386
MAP2K2	-0.02	-0.47	0.43	0.99	0.72	1.35	0.9283

LILRB3	-0.02	-0.58	0.54	0.99	0.67	1.45	0.9412
HLA-DMB	-0.02	-0.41	0.37	0.99	0.75	1.29	0.9095
FOS	-0.02	-0.51	0.47	0.99	0.70	1.39	0.9276
CD244	-0.02	-0.66	0.62	0.98	0.63	1.53	0.9355
CXCR2	-0.03	-0.59	0.54	0.98	0.66	1.45	0.9215
ALOX 5	-0.03	-0.41	0.36	0.98	0.75	1.28	0.8843
KLRK1	-0.03	-0.75	0.70	0.98	0.59	1.62	0.9374
ITGAX	-0.03	-0.51	0.45	0.98	0.70	1.37	0.9010
CREB5	-0.03	-0.58	0.52	0.98	0.67	1.43	0.9093
IL32	-0.03	-0.50	0.44	0.98	0.71	1.35	0.8921
ATF2	-0.03	-0.39	0.32	0.98	0.76	1.25	0.8430
CD74	-0.04	-0.45	0.38	0.98	0.73	1.30	0.8557
CD40LG	-0.04	-0.58	0.51	0.98	0.67	1.42	0.8867
SMAD3	-0.04	-0.41	0.33	0.97	0.75	1.26	0.8314
IRF2	-0.04	-0.38	0.30	0.97	0.77	1.23	0.8105
HLA-E	-0.04	-0.44	0.36	0.97	0.74	1.28	0.8277
CD96	-0.04	-0.48	0.39	0.97	0.72	1.31	0.8318
LAIR2	-0.05	-0.54	0.45	0.97	0.69	1.37	0.8443
CD4	-0.05	-0.51	0.42	0.97	0.70	1.34	0.8316
TYK2	-0.05	-0.61	0.52	0.97	0.66	1.43	0.8595
JAK3	-0.05	-0.63	0.54	0.97	0.64	1.45	0.8643
PAFAH2	-0.05	-0.93	0.83	0.97	0.53	1.78	0.9092
S100A7	-0.05	-0.91	0.82	0.97	0.53	1.76	0.9070
ADA	-0.05	-0.48	0.39	0.97	0.72	1.31	0.8155
SERPING1	-0.05	-0.89	0.79	0.97	0.54	1.73	0.8982
TXNIP	-0.05	-0.47	0.37	0.97	0.72	1.29	0.7962
ILF3	-0.05	-0.44	0.34	0.96	0.74	1.27	0.7803
HRH2	-0.05	-0.47	0.37	0.96	0.72	1.29	0.7913
TNFRSF1A	-0.05	-0.51	0.40	0.96	0.70	1.32	0.8040
LCN2	-0.05	-0.86	0.75	0.96	0.55	1.68	0.8887
FCER2	-0.05	-0.81	0.70	0.96	0.57	1.63	0.8810
CD47	-0.05	-0.42	0.31	0.96	0.75	1.24	0.7571
XCL2	-0.06	-0.70	0.58	0.96	0.62	1.50	0.8477
STAT2	-0.06	-0.44	0.31	0.96	0.74	1.24	0.7134
TRAF6	-0.07	-0.63	0.50	0.96	0.65	1.41	0.8035
TNFSF8	-0.07	-0.53	0.39	0.95	0.69	1.31	0.7553

PECAM1	-0.07	-0.47	0.33	0.95	0.72	1.26	0.7082
FLT3LG	-0.07	-0.52	0.37	0.95	0.70	1.29	0.7276
CD79B	-0.08	-0.52	0.36	0.95	0.70	1.29	0.7174
CD37	-0.08	-0.63	0.47	0.95	0.65	1.39	0.7673
HLA-DOB	-0.08	-0.48	0.33	0.95	0.72	1.25	0.6836
LILRA5	-0.08	-0.47	0.31	0.95	0.72	1.24	0.6707
IFNGR1	-0.08	-0.47	0.31	0.95	0.72	1.24	0.6702
CFP	-0.08	-0.41	0.25	0.95	0.75	1.19	0.6132
MEF2C	-0.08	-0.43	0.27	0.95	0.74	1.21	0.6290
HAVCR2	-0.08	-0.55	0.38	0.94	0.68	1.30	0.7035
LILRB2	-0.09	-0.56	0.38	0.94	0.68	1.30	0.6917
CXCR3	-0.09	-0.67	0.49	0.94	0.63	1.41	0.7454
CASP3	-0.09	-0.57	0.38	0.94	0.67	1.30	0.6813
IL5RA	-0.09	-0.79	0.60	0.94	0.58	1.52	0.7756
HLA-B	-0.09	-0.45	0.27	0.94	0.73	1.20	0.5815
CD99	-0.09	-0.55	0.36	0.94	0.68	1.29	0.6603
IL16	-0.10	-0.49	0.29	0.93	0.71	1.22	0.5872
MAP3K7	-0.10	-0.57	0.37	0.93	0.67	1.29	0.6511
CCR2	-0.10	-0.53	0.33	0.93	0.69	1.25	0.6185
CD5	-0.10	-0.57	0.37	0.93	0.67	1.29	0.6399
STAT1	-0.10	-0.56	0.35	0.93	0.68	1.27	0.6269
OAS3	-0.11	-0.62	0.41	0.93	0.65	1.33	0.6636
DOCK9	-0.11	-0.55	0.34	0.93	0.68	1.26	0.6096
CD7	-0.11	-0.66	0.45	0.93	0.63	1.36	0.6823
IL12RB1	-0.11	-0.71	0.49	0.93	0.61	1.40	0.7011
ATG7	-0.11	-0.53	0.31	0.93	0.69	1.24	0.5746
ITGA2B	-0.11	-0.93	0.70	0.92	0.52	1.63	0.7682
IRF8	-0.12	-0.73	0.50	0.92	0.60	1.41	0.6878
TLR8	-0.12	-0.58	0.35	0.92	0.67	1.27	0.5927
IL2RG	-0.12	-0.62	0.38	0.92	0.65	1.30	0.6087
RORA	-0.12	-0.52	0.27	0.92	0.70	1.20	0.5024
CR1	-0.12	-0.58	0.33	0.92	0.67	1.26	0.5635
CXCR6	-0.13	-0.81	0.55	0.92	0.57	1.47	0.6949
CD164	-0.13	-0.47	0.22	0.92	0.72	1.16	0.4374
ENTPD1	-0.13	-0.66	0.40	0.91	0.63	1.32	0.6036
SLAMF7	-0.13	-0.93	0.66	0.91	0.53	1.58	0.7241

HCK	-0.13	-0.63	0.36	0.91	0.65	1.28	0.5673
HLA-A	-0.14	-0.53	0.26	0.91	0.69	1.20	0.4679
CASP8	-0.14	-0.55	0.27	0.91	0.69	1.21	0.4790
MAPK14	-0.14	-0.50	0.23	0.91	0.71	1.17	0.4231
IFNAR2	-0.14	-0.53	0.25	0.91	0.69	1.19	0.4552
TNFRSF13C	-0.14	-0.90	0.62	0.91	0.54	1.53	0.6927
DUSP6	-0.14	-0.53	0.25	0.91	0.69	1.19	0.4448
TARP	-0.14	-0.65	0.36	0.90	0.64	1.28	0.5461
IDO1	-0.15	-0.81	0.52	0.90	0.57	1.43	0.6429
IL10RA	-0.15	-0.54	0.24	0.90	0.69	1.18	0.4331
IRF4	-0.15	-0.95	0.66	0.90	0.52	1.58	0.7014
INPP5D	-0.15	-0.50	0.21	0.90	0.71	1.16	0.3916
CCND3	-0.15	-0.73	0.43	0.90	0.60	1.35	0.5925
CD14	-0.15	-0.65	0.35	0.90	0.64	1.28	0.5283
TFEB	-0.15	-0.79	0.49	0.90	0.58	1.41	0.6189
SH2D1B	-0.15	-0.53	0.22	0.90	0.69	1.17	0.3966
CT45A1	-0.15	-1.17	0.87	0.90	0.44	1.82	0.7504
GZMB	-0.15	-0.85	0.54	0.90	0.55	1.46	0.6391
REL	-0.16	-0.51	0.20	0.90	0.70	1.15	0.3615
CD180	-0.16	-0.64	0.33	0.90	0.64	1.26	0.5026
HRH4	-0.16	-0.94	0.63	0.90	0.52	1.55	0.6728
GZMH	-0.16	-0.71	0.40	0.90	0.61	1.32	0.5467
TLR9	-0.16	-0.98	0.66	0.90	0.51	1.58	0.6808
TNFSF13	-0.16	-0.84	0.52	0.89	0.56	1.43	0.6171
CD3E	-0.16	-0.58	0.26	0.89	0.67	1.20	0.4191
FCGR1A	-0.17	-1.12	0.78	0.89	0.46	1.72	0.7085
LY9	-0.17	-0.69	0.35	0.89	0.62	1.27	0.4896
MME	-0.17	-0.75	0.41	0.89	0.59	1.33	0.5377
IL18R1	-0.17	-0.90	0.56	0.89	0.54	1.47	0.6175
MS4A1	-0.17	-0.60	0.25	0.89	0.66	1.19	0.3923
CXCL11	-0.17	-1.64	1.29	0.89	0.32	2.45	0.8024
PLA2G6	-0.18	-0.80	0.45	0.89	0.58	1.36	0.5516
PTPRC	-0.18	-0.77	0.42	0.88	0.58	1.34	0.5289
CD84	-0.18	-0.59	0.23	0.88	0.66	1.18	0.3652
IL2RB	-0.18	-0.65	0.29	0.88	0.64	1.22	0.4199
CD97	-0.18	-0.64	0.28	0.88	0.64	1.21	0.4055

TNFRSF8	-0.18	-0.63	0.26	0.88	0.65	1.20	0.3928
PRKCD	-0.18	-0.69	0.32	0.88	0.62	1.25	0.4409
ATG16L1	-0.19	-0.64	0.27	0.88	0.64	1.20	0.3855
BTLA	-0.19	-0.55	0.17	0.88	0.68	1.13	0.2769
NR3C1	-0.19	-0.63	0.25	0.88	0.64	1.19	0.3624
LCP1	-0.19	-0.66	0.27	0.87	0.63	1.21	0.3850
STAT3	-0.20	-0.69	0.30	0.87	0.62	1.23	0.4105
CD81	-0.20	-0.53	0.13	0.87	0.69	1.10	0.2216
CYLD	-0.20	-0.67	0.28	0.87	0.63	1.21	0.3842
TNFRSF1B	-0.20	-0.78	0.38	0.87	0.58	1.31	0.4762
CD79A	-0.20	-0.84	0.45	0.87	0.56	1.36	0.5181
ITGAM	-0.20	-0.64	0.24	0.87	0.64	1.18	0.3434
ICOS	-0.20	-0.70	0.30	0.87	0.61	1.23	0.4004
MAVS	-0.21	-0.61	0.20	0.87	0.66	1.15	0.2867
MAP3K5	-0.21	-0.56	0.14	0.86	0.68	1.10	0.2155
ICAM2	-0.21	-0.80	0.38	0.86	0.57	1.30	0.4498
STAT6	-0.21	-0.67	0.24	0.86	0.63	1.18	0.3317
IL6R	-0.22	-0.75	0.32	0.86	0.59	1.25	0.3981
NFATC3	-0.22	-0.67	0.23	0.86	0.63	1.17	0.3135
NFATC1	-0.22	-0.70	0.25	0.86	0.62	1.19	0.3314
AKT3	-0.22	-0.71	0.27	0.86	0.61	1.21	0.3458
IKBKE	-0.22	-0.62	0.17	0.86	0.65	1.13	0.2450
IFI16	-0.22	-0.59	0.14	0.86	0.66	1.11	0.2113
IFI35	-0.22	-0.84	0.39	0.86	0.56	1.31	0.4457
SELL	-0.23	-0.66	0.20	0.85	0.63	1.15	0.2719
PIK3CG	-0.23	-0.54	0.09	0.85	0.69	1.06	0.1409
MYD88	-0.23	-0.71	0.25	0.85	0.61	1.19	0.3184
ITGA6	-0.23	-0.69	0.23	0.85	0.62	1.17	0.2971
CD28	-0.23	-0.97	0.50	0.85	0.51	1.42	0.5070
SPN	-0.23	-0.78	0.31	0.85	0.58	1.24	0.3676
HLA-C	-0.23	-0.69	0.22	0.85	0.62	1.17	0.2896
MICB	-0.24	-0.62	0.15	0.85	0.65	1.11	0.2121
NFKB1	-0.24	-0.72	0.24	0.85	0.61	1.18	0.3013
NOTCH1	-0.25	-0.73	0.24	0.84	0.60	1.18	0.2977
DDX58	-0.25	-0.75	0.26	0.84	0.59	1.20	0.3102
ZAP70	-0.25	-0.71	0.21	0.84	0.61	1.15	0.2564

CSF2RB	-0.25	-0.94	0.44	0.84	0.52	1.36	0.4446
TGFB1	-0.25	-0.81	0.31	0.84	0.57	1.24	0.3463
IL7R	-0.26	-0.74	0.23	0.84	0.60	1.17	0.2745
TCF7	-0.26	-0.64	0.13	0.84	0.64	1.09	0.1697
PTGS2	-0.26	-0.68	0.15	0.83	0.62	1.11	0.1951
KLRC1	-0.27	-0.79	0.26	0.83	0.58	1.20	0.2891
CD44	-0.27	-0.80	0.26	0.83	0.58	1.20	0.2886
THBS1	-0.27	-1.16	0.62	0.83	0.45	1.54	0.5189
CARD11	-0.27	-0.73	0.18	0.83	0.60	1.14	0.2176
ITGAL	-0.28	-0.72	0.16	0.82	0.61	1.12	0.1926
NOD1	-0.28	-1.16	0.60	0.82	0.45	1.52	0.5039
MAP3K1	-0.28	-0.72	0.16	0.82	0.61	1.11	0.1879
CD6	-0.29	-0.71	0.14	0.82	0.61	1.10	0.1727
GNLY	-0.29	-0.83	0.26	0.82	0.56	1.20	0.2771
CCRL2	-0.29	-1.13	0.56	0.82	0.46	1.47	0.4758
GTF3C1	-0.29	-0.84	0.27	0.82	0.56	1.20	0.2802
HLA-DRB3	-0.29	-0.82	0.24	0.82	0.57	1.18	0.2566
FYN	-0.29	-0.77	0.19	0.82	0.59	1.14	0.2103
CX3CR1	-0.29	-0.67	0.08	0.82	0.63	1.06	0.1177
TAP2	-0.29	-0.77	0.18	0.82	0.59	1.14	0.2065
CD22	-0.30	-0.77	0.17	0.81	0.59	1.13	0.1963
EP300	-0.30	-0.71	0.11	0.81	0.61	1.08	0.1398
IL17RA	-0.30	-0.84	0.24	0.81	0.56	1.18	0.2481
IL6ST	-0.30	-0.91	0.30	0.81	0.53	1.23	0.2992
IL11RA	-0.31	-0.75	0.14	0.81	0.59	1.10	0.1587
MR1	-0.31	-0.87	0.26	0.81	0.55	1.19	0.2574
CD24	-0.31	-1.13	0.51	0.81	0.46	1.42	0.4264
NFKB2	-0.31	-0.82	0.19	0.81	0.57	1.14	0.2048
CREB1	-0.31	-0.74	0.11	0.80	0.60	1.08	0.1341
NLRP3	-0.31	-0.85	0.22	0.80	0.55	1.17	0.2260
JAK1	-0.32	-0.72	0.09	0.80	0.61	1.06	0.1133
KLRG1	-0.33	-0.81	0.15	0.80	0.57	1.11	0.1614
CCL3	-0.33	-1.25	0.58	0.79	0.42	1.50	0.4452
TIGIT	-0.33	-0.76	0.09	0.79	0.59	1.07	0.1137
MEFV	-0.34	-0.86	0.18	0.79	0.55	1.13	0.1794
ITGB2	-0.34	-0.74	0.05	0.79	0.60	1.04	0.0842

TLR10	-0.35	-0.87	0.17	0.78	0.55	1.12	0.1667
CD247	-0.35	-0.77	0.07	0.78	0.59	1.05	0.0913
TICAM2	-0.35	-1.19	0.49	0.78	0.44	1.40	0.3822
LTF	-0.35	-1.35	0.64	0.78	0.39	1.56	0.4538
LAMP2	-0.36	-1.00	0.28	0.78	0.50	1.22	0.2504
ITGB1	-0.36	-0.84	0.13	0.78	0.56	1.09	0.1334
HLA-DQB1	-0.36	-0.88	0.16	0.78	0.54	1.11	0.1539
NOD2	-0.37	-0.98	0.25	0.78	0.51	1.19	0.2233
TNFAIP3	-0.37	-0.83	0.09	0.77	0.56	1.07	0.1070
LCK	-0.37	-0.73	-0.01	0.77	0.60	0.99	0.0460
KLRF1	-0.37	-1.00	0.26	0.77	0.50	1.20	0.2255
SH2D1A	-0.38	-0.89	0.14	0.77	0.54	1.10	0.1359
TAPBP	-0.38	-1.01	0.25	0.77	0.50	1.19	0.2143
CD38	-0.39	-1.48	0.71	0.76	0.36	1.63	0.4566
IKBKB	-0.39	-0.89	0.11	0.76	0.54	1.08	0.1168
IFIH1	-0.40	-0.85	0.06	0.76	0.55	1.04	0.0816
CD86	-0.40	-1.08	0.27	0.76	0.47	1.21	0.2219
TAP1	-0.40	-0.85	0.04	0.76	0.56	1.03	0.0720
ST6GAL1	-0.40	-0.89	0.08	0.76	0.54	1.06	0.0939
RUNX1	-0.41	-1.15	0.34	0.75	0.45	1.26	0.2593
TBX21	-0.42	-0.87	0.04	0.75	0.55	1.03	0.0705
RUNX3	-0.42	-0.91	0.08	0.75	0.53	1.05	0.0905
EWSR1	-0.42	-0.81	-0.03	0.75	0.57	0.98	0.0387
CYBB	-0.42	-1.17	0.32	0.75	0.45	1.25	0.2412
NFATC2	-0.43	-0.88	0.02	0.74	0.54	1.01	0.0573
IRF5	-0.44	-1.12	0.23	0.74	0.46	1.17	0.1801
IKBKG	-0.45	-0.93	0.02	0.73	0.53	1.02	0.0600
BCL6	-0.47	-1.01	0.07	0.72	0.50	1.05	0.0841
BCL2	-0.47	-1.18	0.23	0.72	0.44	1.18	0.1720
ETS1	-0.48	-0.88	-0.08	0.72	0.54	0.95	0.0224
PRF1	-0.49	-0.98	-0.01	0.71	0.51	1.00	0.0475
LRRN3	-0.50	-1.24	0.25	0.71	0.42	1.19	0.1715
IGF1R	-0.50	-1.00	0.00	0.71	0.50	1.00	0.0494
ITK	-0.50	-1.01	0.01	0.71	0.50	1.00	0.0520
ITGB3	-0.51	-1.88	0.86	0.70	0.27	1.81	0.4342
CD8A	-0.53	-1.29	0.23	0.69	0.41	1.17	0.1557

YTHDF2	-0.54	-1.13	0.06	0.69	0.46	1.04	0.0743
NLRC5	-0.54	-1.03	-0.04	0.69	0.49	0.97	0.0359
IFNAR1	-0.54	-1.30	0.22	0.69	0.41	1.17	0.1506
IGF2R	-0.55	-1.24	0.14	0.68	0.42	1.10	0.1094
CMKLR1	-0.55	-1.03	-0.07	0.68	0.49	0.95	0.0289
LRP1	-0.56	-1.54	0.41	0.68	0.34	1.33	0.2304
IL15	-0.57	-1.11	-0.02	0.68	0.46	0.99	0.0433
PTGES2	-0.57	-1.18	0.03	0.67	0.44	1.02	0.0613
HLA-DQA1	-0.58	-1.22	0.06	0.67	0.43	1.04	0.0712
NCR1	-0.61	-1.00	-0.22	0.66	0.50	0.86	0.0053
CLEC4C	-0.63	-1.48	0.22	0.65	0.36	1.17	0.1342
LAMP1	-0.64	-1.35	0.08	0.64	0.39	1.06	0.0764
CYSLTR2	-0.64	-1.47	0.19	0.64	0.36	1.14	0.1190
CXCR5	-0.65	-1.32	0.01	0.64	0.40	1.01	0.0532
C3AR1	-0.66	-1.34	0.02	0.63	0.39	1.01	0.0564
F2RL1	-0.68	-1.59	0.24	0.63	0.33	1.18	0.1354
TXK	-0.69	-1.34	-0.03	0.62	0.39	0.98	0.0417
CD83	-0.70	-1.36	-0.04	0.61	0.39	0.97	0.0394
SLAMF6	-0.71	-1.24	-0.17	0.61	0.42	0.89	0.0133
GZMM	-0.76	-1.32	-0.21	0.59	0.40	0.87	0.0110
TNFRSF10B	-0.88	-1.49	-0.27	0.54	0.36	0.83	0.0084

Supplementary Table S4. Differential gene expression in nasal lysate between responders and non-responders in response to probiotic supplementation.

Gene	Log2 fold change	Lower confidence limit (log2)	Upper confidence limit (log2)	P value
CR2	2.61	-0.28	5.51	0.0731
IFI27	1.38	-0.37	3.12	0.1117
HRH1	1.27	-0.16	2.70	0.0779
SCGB1A1	1.27	-0.20	2.73	0.0840
MERTK	1.24	-0.13	2.61	0.0726
SAA1	1.20	-0.06	2.46	0.0601
CCL17	1.16	-0.22	2.54	0.0924
MUC1	1.09	-0.02	2.21	0.0546
SPINK5	1.03	-0.34	2.40	0.1276
CD19	1.02	-0.89	2.92	0.2704
SH2D1B	1.00	-0.43	2.42	0.1540
APP	0.99	-0.63	2.61	0.2098
SPA17	0.99	-0.58	2.55	0.1983
TJP1	0.98	-0.09	2.05	0.0692
CARD11	0.98	-0.19	2.15	0.0944
ITGA6	0.96	-0.62	2.55	0.2121
CDH1	0.96	-0.21	2.12	0.0988
CD1C	0.95	-0.92	2.82	0.2922
IDO1	0.94	-0.46	2.35	0.1714
ITGA2	0.93	-0.11	1.97	0.0767
LAMP3	0.91	-0.44	2.26	0.1673
DUSP4	0.91	-0.21	2.04	0.1041
NOS2	0.87	-0.41	2.14	0.1669
IL18	0.86	-0.07	1.80	0.0681
CD99	0.84	-0.28	1.96	0.1295
CD9	0.82	-0.30	1.94	0.1370
DOCK9	0.82	-0.42	2.06	0.1789
ISG15	0.81	-0.56	2.17	0.2244
CCL3	0.79	-1.70	3.28	0.5060
TAP1	0.77	-0.54	2.08	0.2256
GTF3C1	0.77	-0.26	1.80	0.1319
RORA	0.75	-0.38	1.89	0.1755
TP53	0.74	-0.59	2.06	0.2538

GZMB	0.73	-1.33	2.78	0.4579
TICAM1	0.72	-0.37	1.82	0.1778
CXCL6	0.71	-0.70	2.12	0.2968
CTSH	0.71	-0.43	1.85	0.2034
FOXJ1	0.69	-0.78	2.17	0.3286
SLPI	0.68	-0.31	1.67	0.1595
PTGES2	0.68	-0.39	1.74	0.1944
CD74	0.66	-0.25	1.57	0.1399
IL5RA	0.66	-1.01	2.33	0.4099
ST6GAL1	0.66	-0.62	1.93	0.2873
MAF	0.65	-0.54	1.84	0.2597
ITGB4	0.65	-0.32	1.62	0.1721
CD59	0.63	-0.33	1.58	0.1813
ANXA1	0.62	-0.23	1.47	0.1371
HLA-DRA	0.61	-0.41	1.64	0.2209
CD81	0.61	-0.79	2.01	0.3656
HLA-DQA1	0.61	-1.14	2.36	0.4660
SERPINB2	0.60	-0.69	1.89	0.3344
IRF8	0.59	-0.76	1.95	0.3620
IL1RL1	0.58	-1.27	2.43	0.5115
BCL2L1	0.58	-0.41	1.56	0.2289
HLA-DPB1	0.57	-1.97	3.12	0.6340
PRKCE	0.56	-0.62	1.75	0.3238
BATF	0.56	-0.48	1.59	0.2659
OAS3	0.55	-0.34	1.45	0.2030
PSMB10	0.55	-0.56	1.66	0.3008
CD22	0.55	-1.39	2.49	0.5504
HLA-DMA	0.55	-0.54	1.64	0.2976
TLR8	0.55	-1.92	3.01	0.6392
RUNX3	0.54	-0.55	1.64	0.3032
ITGB1	0.54	-0.35	1.44	0.2144
C1QB	0.54	-0.60	1.68	0.3285
SMAD3	0.53	-0.70	1.75	0.3706
RRAD	0.52	-1.17	2.22	0.5182
HLA-DRB3	0.52	-0.43	1.48	0.2591
PAFAH2	0.52	-0.67	1.70	0.3615

ETS1	0.51	-0.32	1.34	0.2044
CLDN1	0.51	-0.41	1.43	0.2537
ALCAM	0.51	-0.47	1.49	0.2849
PNMA1	0.49	-0.68	1.67	0.3804
ITGAE	0.49	-0.58	1.57	0.3386
PSMB7	0.49	-0.55	1.53	0.3266
PPBP	0.48	-2.02	2.99	0.6841
CD86	0.46	-1.69	2.60	0.6546
CTSL	0.45	-0.70	1.60	0.4105
NFATC2	0.45	-0.73	1.63	0.4245
NUP107	0.45	-0.47	1.37	0.3133
RUNX1	0.44	-0.74	1.63	0.4351
IRF4	0.43	-1.70	2.57	0.6682
CCL4	0.43	-2.29	3.15	0.7379
S100A8	0.42	-1.26	2.11	0.5964
ARG2	0.41	-0.64	1.47	0.4145
TAP2	0.41	-0.39	1.21	0.2903
STAT2	0.38	-0.58	1.34	0.4125
IFIT1	0.37	-1.38	2.11	0.6574
CARD9	0.34	-0.93	1.60	0.5760
LTB	0.34	-1.84	2.51	0.7436
CD276	0.33	-0.88	1.54	0.5642
CD84	0.33	-1.04	1.71	0.6110
CASP3	0.33	-0.60	1.25	0.4583
IKBKB	0.32	-0.52	1.16	0.4298
SERPING1	0.31	-1.56	2.18	0.7259
MAVS	0.31	-0.67	1.28	0.5092
REPS1	0.30	-0.70	1.31	0.5239
IL1RN	0.30	-1.33	1.93	0.6933
FLT3LG	0.30	-0.84	1.43	0.5854
NT5E	0.29	-0.99	1.57	0.6331
ABL1	0.28	-0.78	1.33	0.5827
CT45A1	0.27	-2.32	2.87	0.8232
CCND3	0.26	-0.53	1.04	0.4964
LAMP1	0.26	-0.57	1.08	0.5167
PLA2G6	0.25	-0.81	1.32	0.6170

MAPK3	0.25	-0.57	1.06	0.5231
TNFRSF18	0.24	-0.82	1.30	0.6294
RPS6	0.24	-0.63	1.12	0.5632
TNFRSF12A	0.24	-1.21	1.69	0.7277
BMI1	0.24	-0.97	1.45	0.6767
MFGE8	0.24	-0.78	1.25	0.6260
COL3A1	0.23	-2.78	3.25	0.8692
MX1	0.23	-0.65	1.12	0.5760
BLNK	0.23	-0.80	1.26	0.6330
IFNA7	0.23	-1.65	2.11	0.7950
MRC1	0.22	-1.27	1.71	0.7512
IRF3	0.22	-1.23	1.68	0.7458
CCL2	0.22	-2.03	2.48	0.8356
TNFSF10	0.22	-0.71	1.15	0.6196
ENG	0.20	-1.30	1.70	0.7774
YTHDF2	0.20	-0.72	1.12	0.6473
PVR	0.20	-0.76	1.15	0.6665
PSMD7	0.19	-0.70	1.08	0.6502
CX3CL1	0.19	-1.37	1.75	0.7958
C1QBP	0.19	-1.02	1.40	0.7431
EPCAM	0.19	-0.75	1.13	0.6763
PIK3CG	0.18	-1.31	1.67	0.7948
LRP1	0.18	-1.17	1.52	0.7795
CD63	0.17	-0.61	0.95	0.6528
NOS2A	0.16	-0.91	1.22	0.7563
TSC22D3	0.16	-1.23	1.54	0.8112
MAP3K1	0.16	-0.70	1.01	0.7018
CD40	0.15	-0.84	1.15	0.7448
CD38	0.15	-1.78	2.09	0.8673
HLA-DOB	0.13	-1.44	1.70	0.8589
TRAF6	0.13	-0.83	1.09	0.7734
MICA	0.12	-1.05	1.30	0.8234
CD46	0.12	-0.81	1.05	0.7791
TLR9	0.12	-1.83	2.07	0.8947
PTGDR2	0.12	-2.40	2.63	0.9209
DUSP6	0.12	-1.14	1.38	0.8445

IKBKE	0.12	-0.91	1.14	0.8128
ELK1	0.11	-0.87	1.08	0.8192
ANP32B	0.10	-0.74	0.93	0.8059
IRF7	0.10	-1.16	1.36	0.8721
PDGFC	0.09	-0.96	1.15	0.8503
CFI	0.09	-0.94	1.11	0.8563
SLAMF7	0.09	-1.48	1.66	0.9066
CCL26	0.08	-1.58	1.74	0.9224
PSEN2	0.07	-0.74	0.89	0.8481
IL32	0.07	-1.31	1.44	0.9153
HLA-DPA1	0.06	-1.17	1.29	0.9200
ATG16L1	0.05	-0.99	1.09	0.9130
HMGB1	0.04	-1.39	1.46	0.9559
ENTPD1	0.04	-1.66	1.73	0.9640
S100A7	0.03	-1.88	1.93	0.9756
ATF2	0.02	-0.84	0.89	0.9572
IL6ST	0.02	-0.88	0.92	0.9601
HLA-DRB4	0.02	-2.99	3.03	0.9892
HLA-DQB1	0.02	-1.61	1.65	0.9817
MAP2K2	0.01	-0.78	0.80	0.9793
PIN1	0.00	-0.87	0.87	0.9983
CCR3	-0.01	-2.25	2.23	0.9913
ILF3	-0.01	-0.80	0.78	0.9718
CCL28	-0.02	-1.58	1.54	0.9814
CDKN1A	-0.02	-1.04	0.99	0.9659
SMAD2	-0.02	-2.15	2.10	0.9801
GPI	-0.03	-1.13	1.07	0.9571
APOE	-0.03	-1.79	1.72	0.9692
TNF	-0.04	-2.33	2.24	0.9683
CD47	-0.05	-0.85	0.75	0.8952
IL19	-0.06	-1.70	1.58	0.9385
IFIH1	-0.06	-1.09	0.96	0.8990
UBC	-0.06	-0.98	0.86	0.8869
HAVCR2	-0.07	-1.55	1.42	0.9257
C2	-0.07	-1.24	1.10	0.9007
BCL10	-0.07	-0.87	0.73	0.8521

TIRAP	-0.08	-0.86	0.71	0.8327
MIF	-0.08	-1.18	1.02	0.8755
IL7R	-0.10	-1.89	1.70	0.9103
EWSR1	-0.10	-0.89	0.69	0.7942
FCER1A	-0.10	-1.32	1.11	0.8604
PSEN1	-0.11	-0.97	0.75	0.7916
TFRC	-0.13	-1.12	0.86	0.7823
TNFSF13	-0.13	-1.13	0.87	0.7821
S100A12	-0.13	-2.28	2.02	0.8965
MAPK8	-0.14	-1.04	0.75	0.7398
IGF1R	-0.15	-1.04	0.75	0.7292
CD83	-0.15	-2.51	2.22	0.8958
RIPK2	-0.15	-1.69	1.39	0.8378
TAPBP	-0.15	-0.98	0.68	0.7037
IL23A	-0.15	-1.95	1.65	0.8608
TRAF3	-0.15	-1.17	0.87	0.7542
IL18R1	-0.15	-1.35	1.05	0.7890
CD36	-0.16	-2.05	1.73	0.8609
PSMB9	-0.16	-1.24	0.92	0.7585
CCL3L1	-0.16	-2.72	2.40	0.8952
DDX58	-0.16	-1.18	0.87	0.7429
C3	-0.17	-1.31	0.96	0.7510
IL10RA	-0.18	-2.45	2.08	0.8651
CCL20	-0.18	-3.12	2.76	0.8952
PSMB8	-0.18	-0.95	0.58	0.6129
NLRP3	-0.19	-2.90	2.53	0.8854
ITCH	-0.19	-1.17	0.80	0.6881
IRAK4	-0.19	-1.16	0.78	0.6779
JUN	-0.20	-1.75	1.36	0.7895
CYSLTR1	-0.20	-1.86	1.47	0.8009
MR1	-0.21	-1.30	0.88	0.6834
VEGFA	-0.22	-1.48	1.05	0.7206
MST1R	-0.22	-1.23	0.79	0.6477
LTK	-0.23	-1.79	1.32	0.7506
BST2	-0.23	-1.75	1.29	0.7444
IL13RA1	-0.24	-1.20	0.72	0.6040

IL4R	-0.24	-1.55	1.07	0.6961
ADORA2A	-0.25	-2.90	2.40	0.8443
IRF1	-0.25	-1.48	0.98	0.6672
IL15	-0.26	-1.73	1.22	0.7153
BAX	-0.26	-1.00	0.49	0.4722
MAP2K4	-0.26	-1.12	0.60	0.5262
CR1	-0.26	-3.00	2.48	0.8402
IFNAR1	-0.27	-1.29	0.76	0.5865
VEGFC	-0.27	-1.36	0.82	0.6013
ECSIT	-0.29	-1.30	0.72	0.5520
IL3RA	-0.29	-2.26	1.69	0.7597
CD4	-0.29	-1.63	1.04	0.6454
EDN1	-0.29	-2.70	2.11	0.7974
HLA-DMB	-0.30	-1.25	0.66	0.5159
JAK2	-0.30	-1.17	0.57	0.4708
KIT	-0.31	-1.60	0.99	0.6194
MYD88	-0.31	-1.36	0.74	0.5379
MICB	-0.32	-1.59	0.96	0.6031
NR3C1	-0.32	-1.29	0.66	0.4956
BID	-0.32	-2.30	1.65	0.7279
F13A1	-0.33	-2.44	1.78	0.7442
CYLD	-0.33	-1.41	0.75	0.5232
CXCL1	-0.33	-2.55	1.88	0.7504
ATF1	-0.34	-1.23	0.56	0.4320
C3AR1	-0.34	-2.76	2.08	0.7679
ATG7	-0.34	-1.67	0.98	0.5880
SPN	-0.35	-2.36	1.66	0.7110
TRAF2	-0.36	-1.44	0.73	0.4919
MAPK1	-0.36	-1.27	0.56	0.4128
HRH2	-0.36	-2.60	1.88	0.7340
LILRB3	-0.36	-2.83	2.10	0.7558
CD58	-0.37	-1.50	0.76	0.4962
NRP1	-0.37	-1.78	1.04	0.5789
EGR2	-0.37	-2.41	1.67	0.6986
C1QA	-0.38	-1.69	0.93	0.5451
IRAK2	-0.38	-1.75	0.99	0.5628

CYBB	-0.38	-3.11	2.35	0.7690
NFKB1	-0.39	-1.63	0.84	0.5042
STAT3	-0.40	-1.52	0.72	0.4573
HLA-E	-0.40	-1.62	0.82	0.4929
ATG10	-0.40	-1.65	0.85	0.5004
NLRC5	-0.41	-1.37	0.55	0.3726
BCL2	-0.41	-1.65	0.83	0.4856
REL	-0.41	-1.40	0.58	0.3825
IL1R1	-0.42	-1.50	0.66	0.4164
CFD	-0.43	-1.69	0.83	0.4712
IFI35	-0.45	-1.26	0.36	0.2549
BTK	-0.45	-3.25	2.35	0.7352
TBK1	-0.45	-1.44	0.54	0.3409
SIGIRR	-0.45	-1.32	0.42	0.2828
CCL23	-0.46	-2.99	2.08	0.7044
MAP2K1	-0.46	-1.50	0.58	0.3570
MAP3K5	-0.46	-1.36	0.43	0.2849
PYCARD	-0.47	-1.56	0.63	0.3759
IRF2	-0.47	-1.61	0.67	0.3909
EP300	-0.47	-1.43	0.50	0.3136
NFATC3	-0.48	-1.64	0.67	0.3850
JAK1	-0.49	-1.55	0.57	0.3392
NFKBIA	-0.49	-2.23	1.25	0.5552
PTGS1	-0.49	-2.10	1.12	0.5200
MAP3K7	-0.50	-1.38	0.39	0.2459
ICAM1	-0.51	-2.73	1.70	0.6250
CHUK	-0.52	-1.47	0.43	0.2602
RELA	-0.52	-1.60	0.56	0.3205
STAT1	-0.53	-1.55	0.49	0.2830
HLA-C	-0.53	-1.60	0.54	0.3021
SBNO2	-0.53	-1.76	0.70	0.3681
TYK2	-0.53	-1.40	0.33	0.2075
ATG5	-0.54	-1.36	0.29	0.1820
FCGR2B	-0.54	-2.62	1.54	0.5853
THBD	-0.54	-2.65	1.57	0.5901
CXCR1	-0.54	-3.22	2.14	0.6700

NFATC1	-0.54	-1.51	0.42	0.2435
SPP1	-0.54	-2.28	1.19	0.5102
CD274	-0.55	-1.72	0.62	0.3304
HLA-A	-0.55	-1.58	0.47	0.2670
LILRA1	-0.55	-2.96	1.85	0.6275
TXNIP	-0.57	-1.73	0.59	0.3077
IL33	-0.57	-2.30	1.16	0.4877
NFATC4	-0.58	-2.14	0.98	0.4404
LTBR	-0.58	-1.45	0.29	0.1754
CD68	-0.58	-1.82	0.66	0.3337
SYK	-0.58	-1.80	0.64	0.3218
F2RL1	-0.58	-1.46	0.29	0.1740
IRAK1	-0.59	-1.73	0.55	0.2845
IL1A	-0.60	-3.05	1.86	0.6090
IFNAR2	-0.60	-1.60	0.41	0.2221
IKBKG	-0.61	-1.52	0.30	0.1728
STAT6	-0.62	-1.66	0.43	0.2251
IFI16	-0.62	-1.46	0.21	0.1317
CREB1	-0.63	-1.46	0.20	0.1264
TNFRSF8	-0.63	-2.89	1.63	0.5592
CASP8	-0.63	-1.92	0.66	0.3091
IL2RA	-0.63	-2.42	1.16	0.4592
CD48	-0.64	-2.58	1.31	0.4923
TAB1	-0.64	-1.64	0.36	0.1886
ALOX 5	-0.64	-2.68	1.40	0.5078
TOLLIP	-0.65	-1.55	0.25	0.1410
HLA-B	-0.67	-1.82	0.47	0.2248
TLR1	-0.68	-2.30	0.93	0.3782
IL16	-0.69	-2.62	1.23	0.4520
NFKB2	-0.70	-2.26	0.87	0.3545
FCGR1A	-0.70	-2.66	1.26	0.4538
IL2RG	-0.71	-2.70	1.29	0.4587
TLR3	-0.72	-1.77	0.34	0.1682
NOD2	-0.75	-2.29	0.80	0.3164
ITGAL	-0.75	-2.86	1.37	0.4596
TPSAB1	-0.75	-2.75	1.25	0.4347

HRAS	-0.76	-1.79	0.28	0.1389
LILRB2	-0.77	-3.20	1.65	0.5045
CLEC4A	-0.77	-2.82	1.27	0.4301
CD97	-0.79	-2.77	1.19	0.4061
CXCL16	-0.79	-1.88	0.30	0.1430
PPARG	-0.80	-2.12	0.52	0.2151
AMICA1	-0.80	-3.24	1.63	0.4895
ITGB2	-0.81	-2.89	1.28	0.4176
LAMP2	-0.82	-1.91	0.27	0.1293
MAPK14	-0.82	-1.89	0.24	0.1198
FPR2	-0.84	-3.44	1.77	0.5000
TFEB	-0.84	-2.23	0.55	0.2133
LTB4R	-0.84	-2.38	0.69	0.2567
PLAU	-0.84	-2.64	0.95	0.3288
CTSS	-0.85	-2.35	0.66	0.2465
RORC	-0.85	-2.14	0.45	0.1812
BCL6	-0.85	-2.19	0.48	0.1916
TNFRSF1A	-0.87	-2.29	0.55	0.2101
TNFSF12	-0.87	-2.15	0.41	0.1661
CDK1	-0.87	-2.66	0.91	0.3087
CXCR4	-0.88	-3.65	1.88	0.5028
THBS1	-0.90	-3.34	1.54	0.4389
CD53	-0.91	-3.52	1.70	0.4661
LYN	-0.91	-2.33	0.50	0.1864
CSF3R	-0.92	-3.39	1.56	0.4371
MAPKAPK2	-0.92	-2.07	0.23	0.1072
SELPLG	-0.93	-3.21	1.35	0.3951
HCK	-0.93	-3.31	1.45	0.4144
AXL	-0.94	-2.27	0.39	0.1516
DEFB1	-0.94	-1.93	0.05	0.0605
TGFB1	-0.95	-2.97	1.08	0.3314
CD55	-0.96	-2.44	0.53	0.1871
PRKCD	-0.96	-2.37	0.46	0.1677
SOCS1	-0.96	-2.73	0.81	0.2628
TREM1	-0.97	-3.57	1.63	0.4363
ITGAM	-0.97	-3.32	1.37	0.3874

ICAM4	-0.98	-3.42	1.47	0.4043
TNFRSF10B	-1.00	-2.19	0.20	0.0940
SLC11A1	-1.01	-3.63	1.61	0.4204
PTPRC	-1.02	-3.66	1.62	0.4199
FCER1G	-1.04	-3.30	1.22	0.3400
TNFRSF14	-1.04	-2.18	0.11	0.0718
JAK3	-1.04	-3.15	1.06	0.3044
CCRL2	-1.06	-3.34	1.23	0.3372
IFNGR1	-1.06	-2.21	0.09	0.0686
PTGS2	-1.06	-3.95	1.82	0.4417
TNFAIP3	-1.07	-3.26	1.12	0.3112
PECAM1	-1.07	-3.57	1.43	0.3734
IL15RA	-1.07	-2.22	0.08	0.0648
ISG20	-1.09	-2.55	0.36	0.1293
INPP5D	-1.11	-3.04	0.82	0.2360
LY96	-1.12	-3.53	1.29	0.3346
TANK	-1.13	-2.73	0.48	0.1547
NCR1	-1.13	-4.16	1.91	0.4366
CCR1	-1.13	-3.74	1.48	0.3670
STAT5B	-1.14	-2.82	0.53	0.1650
EGR1	-1.14	-2.59	0.30	0.1100
CKLF	-1.16	-3.09	0.77	0.2167
LCP1	-1.16	-3.67	1.34	0.3346
NOTCH1	-1.17	-2.73	0.40	0.1314
TLR2	-1.18	-3.69	1.34	0.3319
CD37	-1.18	-3.49	1.14	0.2924
SH2B2	-1.18	-3.39	1.03	0.2712
TNFRSF1B	-1.19	-3.90	1.52	0.3610
IFITM1	-1.19	-3.62	1.24	0.3109
ICAM3	-1.19	-3.27	0.88	0.2369
MAP4K2	-1.20	-2.62	0.22	0.0914
TLR4	-1.22	-3.75	1.31	0.3172
CD44	-1.23	-2.84	0.38	0.1217
PIK3CD	-1.24	-3.55	1.07	0.2675
CSF2RB	-1.24	-3.91	1.42	0.3327
OSM	-1.25	-3.98	1.48	0.3419

FUT7	-1.27	-3.74	1.19	0.2854
RELB	-1.28	-2.84	0.29	0.1015
CREB5	-1.28	-3.54	0.98	0.2443
TNFRSF10C	-1.28	-3.57	1.00	0.2474
IL17RA	-1.29	-3.09	0.52	0.1483
TNFSF15	-1.29	-3.96	1.38	0.3149
CD14	-1.30	-3.34	0.74	0.1923
LIF	-1.32	-3.07	0.43	0.1277
CXCL3	-1.32	-3.09	0.45	0.1309
CLEC7A	-1.32	-3.46	0.81	0.2036
LTF	-1.32	-3.22	0.57	0.1552
BST1	-1.33	-3.11	0.45	0.1304
CXCR2	-1.33	-3.61	0.95	0.2295
ITGA5	-1.35	-3.64	0.95	0.2280
IFITM2	-1.37	-3.72	0.98	0.2301
TNFSF13B	-1.37	-3.58	0.83	0.2022
TFE3	-1.39	-2.85	0.07	0.0598
NCF4	-1.41	-4.15	1.33	0.2858
CFP	-1.42	-3.49	0.65	0.1626
IL1R2	-1.43	-3.82	0.96	0.2190
IL1RAP	-1.43	-3.32	0.45	0.1238
TNFSF14	-1.45	-4.13	1.22	0.2611
CEBPB	-1.46	-4.27	1.35	0.2824
MEFV	-1.47	-4.26	1.31	0.2743
ITGAX	-1.50	-4.16	1.16	0.2458
IFIT2	-1.51	-3.47	0.44	0.1184
MME	-1.51	-4.35	1.32	0.2701
IL6R	-1.53	-3.82	0.77	0.1745
LILRA5	-1.53	-3.89	0.83	0.1848
CYFIP2	-1.54	-3.51	0.42	0.1136
CD164	-1.55	-4.82	1.72	0.3259
PLAUR	-1.55	-4.38	1.28	0.2590
CXCL2	-1.56	-4.62	1.49	0.2887
IL8	-1.63	-4.73	1.48	0.2790
CASP1	-1.64	-3.74	0.46	0.1155
SELL	-1.70	-3.71	0.31	0.0900

TLR6	-1.73	-3.72	0.27	0.0848
IL1B	-1.77	-4.30	0.76	0.1549
FCGR2A	-1.77	-4.14	0.59	0.1296
FCGR3A	-2.11	-5.31	1.09	0.1779
IL18RAP	-2.21	-5.18	0.76	0.1323
DMBT1	2.68	0.28	5.08	0.0312
TARP	2.26	0.50	4.03	0.0156
TNFRSF11A	1.51	0.21	2.81	0.0260
CEACAM1	1.35	0.13	2.57	0.0330
TGFB2	1.31	0.12	2.50	0.0332
CFB	1.31	0.00	2.61	0.0497
CEACAM6	1.19	0.13	2.26	0.0303
LGALS3	1.06	0.05	2.08	0.0417
LCN2	1.06	0.02	2.10	0.0470
CD24	0.92	0.02	1.82	0.0456
IGF2R	-1.48	-2.93	-0.04	0.0451
FOS	-1.95	-3.49	-0.41	0.0170

Supplementary Table S5. List of 30 additional genes added to PanCancer Immune Profiling panel.

NCBI reference sequence	Official gene symbol	Gene name
NM_021101.4	CLDN1	Claudin 1
NM_001301025.1	TJP1	tight junction protein 1
NM_001205254.1	OCLN	Occludin
NM_002016.1	FLG	Filaggrin
NM_005201.3	CCR8	Chemokine (C-C motif) receptor 8
NM_002619.3	PF4	Platelet factor 4
NM_033035.4	TSLP	Thymic stromal lymphopoietin
NM_000437.3	PAFAH2	platelet activating factor acetylhydrolase 2
NM_000861.3	HRH1	Histamine receptor H1
NM_001131055.1	HRH2	Histamine receptor H2
NM_007232.2	HRH3	Histamine receptor H3
NM_001143828.1	HRH4	Histamine receptor H4
NM_000176.2	NR3C1	nuclear receptor subfamily 3 group C member 1
NM_001168319.1	EDN1	Endothelin 1
NM_001302455.1	EDN3	Endothelin 3
NM_002935.2	RNASE3	ribonuclease A family member 3
NM_000625.4	NOS2	nitric oxide synthase 2
NM_003182.2	TAC1	tachykinin, precursor 1
NM_001015881.1	TSC22D3	TSC22 domain family member 3
NM_002228.3	JUN	Jun proto-oncogene
NM_003064.3	SLPI	secretory leukocyte peptidase inhibitor
NM_001178130.2	EGF	epidermal growth factor
NM_000962.3	PTGS1	prostaglandin-endoperoxide synthase 1
NM_003357.4	SCGB1A1	secretoglobin family 1A member 1
NM_001282186.1	CYSLTR1	cysteinyl leukotriene receptor 1
NM_001308465.1	CYSLTR2	cysteinyl leukotriene receptor 2
NM_000698.2	ALOX5	arachidonate 5-lipoxygenase
NM_001282601.1	PTGES2	prostaglandin E synthase 2
NM_001143919.2	LTB4R	leukotriene B4 receptor
NM_001199640.1	IL33	interleukin 33
